# A systematic review investigating measurement properties of physiological tests in rugby

**DOI:** 10.1186/s13102-017-0081-1

**Published:** 2017-12-28

**Authors:** Matthew Chiwaridzo, Sander Oorschot, Jermaine M. Dambi, Gillian D. Ferguson, Emmanuel Bonney, Tapfuma Mudawarima, Cathrine Tadyanemhandu, Bouwien C. M. Smits-Engelsman

**Affiliations:** 1 Department of Health and Rehabilitation Sciences, Division of Physiotherapy, Faculty of Health Sciences, University of Cape Town, Cape Town, South Africa; 2 Department of Human Movement Sciences, Univeristy of Maastricht, Faculty of Health, Medicine and Life Sciences, Maastricht, Netherlands; 3 Rehabilitation Department, University of Zimbabwe, College of Health Sciences, P.O Box A178, Avondale, Harare, Zimbabwe; 4 Department of Physiotherapy, University of Ghana, College of Health Sciences, School of Biomedical and Allied Health Sciences, Accra, Ghana; 5 Harare Central Hospital, Rehabilitation Department, P.O Box ST 14, Southerton, Lobengula Road, Harare, Zimbabwe; 6 Department of Physiotherapy, University of Witwatersrand, Faculty of Health Sciences, School of Therapeutic Sciences, 7 York Road, Parktown, Johannesburg, South Africa

**Keywords:** Reliability, Validity, Responsiveness, Physiological characteristics, Rugby, Systematic review

## Abstract

**Background:**

This systematic review was conducted with the first objective aimed at providing an overview of the physiological characteristics commonly evaluated in rugby and the corresponding tests used to measure each construct. Secondly, the measurement properties of all identified tests per physiological construct were evaluated with the ultimate purpose of identifying tests with strongest level of evidence per construct.

**Methods:**

The review was conducted in two stages. In all stages, electronic databases of EBSCOhost, Medline and Scopus were searched for full-text articles. Stage 1 included studies examining physiological characteristics in rugby. Stage 2 included studies evaluating measurement properties of all tests identified in Stage 1 either in rugby or related sports such as Australian Rules football and Soccer. Two independent reviewers screened relevant articles from titles and abstracts for both stages.

**Results:**

Seventy studies met the inclusion criteria for Stage 1. The studies described 63 tests assessing speed (8), agility/change of direction speed (7), upper-body muscular endurance (8), upper-body muscular power (6), upper-body muscular strength (5), anaerobic endurance (4), maximal aerobic power (4), lower-body muscular power (3), prolonged high-intensity intermittent running ability/endurance (5), lower-body muscular strength (5), repeated high-intensity exercise performance (3), repeated-sprint ability (2), repeated-effort ability (1), maximal aerobic speed (1) and abdominal endurance (1). Stage 2 identified 20 studies describing measurement properties of 21 different tests. Only moderate evidence was found for the reliability of the 30–15 Intermittent Fitness. There was limited evidence found for the reliability and/or validity of 5 m, 10 m, 20 m speed tests, 505 test, modified 505 test, L run test, Sergeant Jump test and bench press repetitions-to-fatigue tests. There was no information from high-quality studies on the measurement properties of all the other tests identified in stage 1.

**Conclusion:**

A number of physiological characteristics are evaluated in rugby. Each physiological construct has multiple tests for measurement. However, there is paucity of information on measurement properties from high-quality studies for the tests. This raises questions about the usefulness and applicability of these tests in rugby and creates a need for high-quality future studies evaluating measurement properties of these physiological tests.

**Trial registrations:**

PROSPERO CRD 42015029747.

**Electronic supplementary material:**

The online version of this article (10.1186/s13102-017-0081-1) contains supplementary material, which is available to authorized users.

## Background

Rugby (either rugby union or league) is a popular sport played professionally or otherwise at both junior and senior levels worldwide [1]. It is generally considered a physical sport characterised by multiple high-intensity activities interspersed with low-intensity activities [2–5]. The players engage in physically demanding contests such as tackles, rucks and mauls with the primary objective of gaining possession of the ball [6]. These contests require players to possess a wide range of physiological characteristics such as strength, power and endurance which allows them to be stronger and fatigue-resistant [7–10].

There are numerous studies in the literature that have provided scientific evidence on the physiological characteristics of rugby players. This has been necessitated by the drive to understand the physiological factors that differentiate between playing levels (talent identification) and the physiological characteristics associated with optimal performance [1, 2, 7, 10–18]. For example, Gabbett and Seibold [15] postulated that lower body power, upper-body strength-endurance, and prolonged high-intensity intermittent running ability discriminated players for team selection in semi-professional rugby league (RL) players. Smart et al. [17] found correlations between speed, repeated- sprint ability and game performance statistics such as tackle breaks and tries scored in rugby union (RU). Furthermore, Till et al. [18] compared longitudinal changes in physical qualities with career attainment status and found that advanced physical qualities such as absolute strength during the adolescence period contributed significantly to the attainment of professional status in rugby. All these findings suggest an important relationship between physiological characteristics and future career success, physical performance and team selection [15, 17, 18].

Today, physiological profiling of rugby players has become an integral aspect of the contemporary sport of rugby. It allows coaches to determine “competent” players with enhanced physiological capacities to withstand the high-intensity demands of the sport and can win trophies for team, club or country [6, 7]. This forms the hallmark of talent identification programmes. Secondly, understanding the physiological qualities needed in the sport of rugby may specifically inform training development practices of future professional players [18]. With the surge in physiological profiling, proliferation of talent identification and development programmes for young rugby players [18], there is need for identification and use of physical tests with known measurement properties (reliability, validity and responsiveness). A scoping review of the literature showed that there are multiple tests available for measuring the same physiological characteristic. For example, agility is a fundamental physiological characteristic required for optimal performance by rugby players. The construct has been evaluated using different tests such as ‘L’ run, Illinois agility run test, agility 505 test, modified 505 test and change of direction speed test in the literature [6, 10, 16, 18–22]. In an attempt to understand the basis of selecting tests, it may be important to have an overview of all the tests that measures a specific physiological construct and evaluate systematically the measurement properties of the identified tests in an attempt to identify test(s) with the strongest level of evidence per construct. Possibly, this information can help us understand the reasons for selection of particular tests for the measurement of a specific physiological characteristic in terms of measurement properties. To our knowledge, there is no systematic review that has been conducted to provide such information. Therefore, this systematic review was conducted with the aim of addressing the following research questions:

1. What physiological characteristics of rugby players are evaluated in the literature and which tests are used to measure each identified characteristic?
2. What is known about the measurement properties (reliability, validity and responsiveness) of each identified physiological test in the sport of rugby? If there is no information on the measurement properties for each test in rugby, is there any evidence available from other closely-related intermittent, collision team sports to rugby such as Australian Rules football, American football or Soccer? In case of multiple tests measuring the same construct, which test(s) has the strongest level of evidence in terms of the measurement properties?

## Stage 1: Methods

This systematic review was registered on PROSPERO with the registration number CRD 42015029747 [21]. This review paper was organised in stages. Stage 1 presents an overview of the physiological characteristics commonly evaluated in rugby and the corresponding tests. Stage 2 presents an overview on the measurement properties of the identified physiological tests. Each stage was written in accordance with the Preferred Reporting Items for Systematic review and Meta-analyses (PRISMA) guidelines by Moher et al. [23].

### Literature search

A literature search was conducted using the following databases: Scopus, Medline via EBSCOhost and via PubMed, Academic Search Premier via EBSCOhost, CINAHL (Cumulative Index of Nursing and Allied Health) via EBSCOhost and Africa-Wide Information via EBSCOhost. The review included studies published in the last 20 years between January 1, 1995, and December 31, 2016. Additionally, a hand search was also conducted on reference lists of selected articles to augment the literature.

### Selection criteria for the studies

#### Sports context

There are two major variants of rugby, namely, RU and RL. Although RU differs significantly from RL in team sizes, scoring and in certain situations of tackling and when the ball goes out, there are striking similarities in game duration, field size, player positions, and goal posts [24]. There are also similarities in the physical demands and physiological responses elicited during game play as both sports are predominantly aerobic in nature interspersed with high-intensity efforts [5, 24]. The objective in both is to get the ball over the opposition’s goal line by carrying, passing, kicking and grounding the ball. Therefore, because of the resemblance we included studies on RU and RL. However, studies on the sport of rugby “sevens” were excluded.

#### Physiological characteristics

Rugby requires a blend of physiological characteristics for players to cope with demands of the game [1]. The studies included had to report on at least one physiological characteristic operationally defined as measures that assess speed, repeated-sprint ability, prolonged high-intensity intermittent running ability, agility, muscular strength, power and endurance and maximal aerobic capacity. In addition, for studies to be included they had to report the name of the test used to measure the physiological construct and include a detailed, reproducible description of the test procedure. There was no restriction in study design applied during study selection. However, editorials, book chapters, poster and oral conference abstracts, unpublished theses, dissertations, and case studies were excluded. Studies published in non-English language were also excluded.

#### Participants

Since rugby is played competitively at junior and senior levels worldwide, studies included in this review had to involve male rugby participants from the age of 10 years and above (adolescents to adults) from any country. Studies involving rugby participants living with disabilities were excluded.

### Search strategy

The search strategy was developed in consultation with an expert librarian in systematic reviews from University of Cape Town (UCT) libraries. The search strategy (see Additional file 1 designed for Medline via PubMed) consisted of a combination of the following search themes connected with the Boolean terms AND:

i. Construct-related general search terms: physical characteristics OR physiological characteristics.
ii. Construct-related specific search terms: speed OR agility OR flexibility.
iii. Target population-related search terms: adult OR adolescent OR youth.
iv. Sport-related search terms: rugby OR rugby union OR rugby league.

### Selection of articles

The selection process was conducted stepwise based on recommendations for performing systematic reviews by van Tulder et al. [25] and Reimers et al. [26]. The first author (MC) ran the search strategy across all databases. Two reviewers (JD and EB) independently reviewed the search results in two steps. The first step involved applying the inclusion criteria to select potentially relevant articles from titles. The abstracts of studies with titles considered relevant were retrieved for further inspection in the second step [26]. Provided that the abstract fulfilled the eligibility criteria or had insufficient information for a selection decision to be made, both reviewers retrieved the full text to further assess for eligibility [26]. Initially, disagreements among reviewers were discussed among themselves at the end of the selection process. In the case of further disagreements, a third (TM) reviewer intervened until a mutual consensus was reached. In addition, all retrieved articles were then reviewed again against the inclusion criteria by the lead investigator (MC).

### Data extraction

Data extraction was performed by two independent people (TM and JD). Extracted data was documented onto a Microsoft Excel data extraction form. The following data were captured for the first objective: publication details of the study (first author, year of publication), the name(s) of the physiological characteristic examined in the study (captured as originally described by the authors) and the name of corresponding test(s) as described in the study used to measure the physiological characteristics. To enable the description of studies, additional information on sport contexts, age of participants, country, target population, study design and sample size were also extracted. The primary author (MC) acted as the data verifier, assessing the exhaustiveness and accuracy of data extracted from the included articles. Discrepancies in data extracted identified by the verifier were communicated to the two data extractors and disagreements resolved by mutual consensus.

## Results: Stage 1

**Fig. 1:**
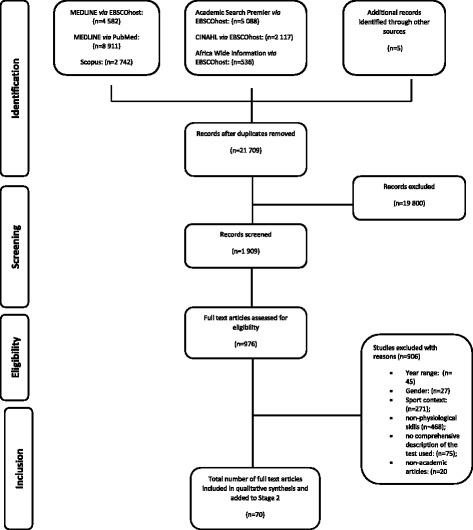
Flow chart of the search and selection process for stage 1 articles

Since Stage 1 results were used to inform the methods and selection criteria for studies in the second stage of the systematic review, results for Stage 1 were presented here. The electronic searches revealed 23,976 studies and after initial selection based on abstract and title, 1909 studies were potentially eligible (Fig. 1). After full-text evaluation, 70 studies were included. The majority of the studies did not meet the inclusion criteria because they did not report on physiological characteristics (Fig. 1).

### Description of included studies

The general characteristics of the 70 included studies are shown in Table 1. Briefly, the majority of the included studies (*n* = 35, 50.0%) were conducted in Australia alone. Only three (4.29%) studies were conducted in an African country, namely, South Africa [7, 27, 28]. Of the 70 studies, 34 (48.6%) had adolescents as participants and six (8.57%) used both adults and adolescents. The sample sizes varied greatly across studies from 12 to 1172 participants depending on study designs. Studies varied from retrospective, prospective cohort studies, experimental with the preponderance of the studies being cross-sectional. The majority of studies (*n* = 50, 71.4%) involved RL participants. Two studies had participants drawn from both RL and RU [24, 29].

**Table 1:** General characteristics of included studies1

Author	Sample size	^ƪ^Age (years)	Target Population	Study design	Country	Sport	Physiological construct
Appleby et al. (2012) [80]	20	24.4 ± 3.4–26.4 ± 3.4	Adults	Longitudinal	Australia	Rugby union	Strength
Argus et al. (2012) [13]	112	16.6 ± 0.8–24.4 ± 2.7	Adolescents & Adults	Cross-sectional	New Zealand	Rugby union	Strength, power
Atkins (2006) [103]	50	21.1 ± 4.7–22.1 ± 5.0	Adults	Cross-sectional	England	Rugby league	^a^HIRA
Austin et al. (2013) [24]	36	24.4 ± 3–24 ± 4	Adults	Test re-test	Australia	Rugby league & union	^b^HIEP
Baker (2009) [81]	64	19.5 ± 1.7–25.0 ± 3.3	Adults	Cross-sectional	Australia	Rugby league	Strength-endurance
Baker and Newton (2008) [77]	40	22.6 ± 3.6–25.3 ± 3.4	Adults	Cross-sectional	Australia	Rugby league	Strength, power, agility, speed.
Baker (2002) [78]	95	16.2 ± 1.2–23.5 ± 3.2	Adolescents & Adults	Cross-sectional	Australia	Rugby league	Strength, power
Bradley et al. (2015) [5]	45	21–33	Adults	Longitudinal (repeated measures)	England	Rugby union	Speed, strength
Comfort et al. (2011) [75]	18	21.7 ± 4.1	Adults	Cross-sectional	England	Rugby league	Speed, agility, power, strength
Cobley et al. (2014) [47]	1172	U13-U15 players	Adolescents	Longitudinal	United Kingdom	Rugby league	Muscular power, speed, change of direction speed, maximal aerobic power
Darrall-Jones et al. (2015) [53]	67	15.5 ± 0.3–19.0 ± 1.1	Adolescents	Cross-sectional	England	Rugby union	Speed, agility, power, ^a^HIRA
Darrall-Jones et al. (2015b) [59]	67	15.4 ± 0.3–19.3 ± 1.2	Adolescents	Cross-sectional	England	Rugby union	Speed, ^a^HIRA, maximal aerobic speed
De Lacey et al. (2014) [104]	39	24 ± 3	Adults	Cross-sectional	New Zealand	Rugby league	Speed, strength, power
Delaney et al. (2015) [72]	31	24.3 ± 4.4	Adults	Cross-sectional	Australia	Rugby league	Speed, change of direction ability, strength, power
Durandt et al. (2014) [27]	174	U16-U18 players	Adolescents	Cross-sectional	South-Africa	Rugby union	Speed, agility, strength, endurance, aerobic fitness
Gabbett (2000) [61]	35	26.5 ± 5.1	Adults	Cross-sectional	Australia	Rugby league	Speed, power, maximal aerobic power
Gabbett (2002a) [30]	159	12.3–25.1	Adolescents & Adults	Cross-sectional	Australia	Rugby league	Power, speed, agility, estimated V0_2MAX_
Meir et al. 2001 [105]	146	N/m	Adults	Cross-sectional	Australia and England	Rugby league	Strength, endurance speed, agility
Gabbett (2005a) [31]	240	16–18	Adolescents	Cross-sectional	Australia	Rugby league	Power, speed, agility, maximal aerobic power
Gabbett (2005b) [32]	45	–	Adolescents	Cross-sectional	Australia	Rugby League	Power, speed, agility, maximal aerobic power
Gabbett (2005c) [33]	68	≥ 18	Adults	Cross-sectional	Australia	Rugby league	Power, speed, agility, maximal aerobic power
Gabbett (2006) [34]	415	21.1 ± 3.4–25.7 ± 5.6	Adults	Cross-sectional	Australia	Rugby league	Power, speed, agility, maximal aerobic power
Gabbett et al. (2007) [35]	86	22.5 ± 4.9	Adults	Cross-sectional	Australia	Rugby league	Power, speed, agility, maximal aerobic power
Gabbett et al. (2008a) [19]	42	23.6 ± 5.3	Adults	Cross-sectional	Australia	Rugby league	Speed, change of direction speed
Gabbett et al. (2008b) [36]	35	14.1 ± 0.2–16.9 ± 0.3	Adolescents	Longitudinal (repeated measures)	Australia	Rugby league	Speed, power, muscular endurance, agility, maximum aerobic power
Gabbett (2009) [73]	12	24.4 ± 3.5	Adults	Cross-sectional	Australia	Rugby league	Acceleration, power, change of direction speed
Gabbett (2009b) [37]	88	13.2 ± 0.6–16.5 ± 0.3	Adolescents	Cross-sectional	Australia	Rugby league	Speed, change of direction speed, power, maximal aerobic power
Gabbett et al. (2011a) [16]	58	23.8 ± 3.8	Adults	Cross-sectional	Australia	Rugby league	Speed, repeated sprint ability, change of direction speed, power, prolonged HIRA
Gabbett et al. (2011b) [49]	86	23.3 ± 3.8	Adults	Cross-sectional	Australia	Rugby league	Speed, change of direction, power, repeated sprint ability, prolonged HIRA, maximal aerobic power
Gabbett et al. (2013) [50]	38	23.1 ± 2.7	Adults	Prospective cohort experimental design	Australia	Rugby league	Repeated sprint ability, prolonged HIRA, maximal aerobic power.
Gabbett et al. (2009c) [65]	64	15.9 ± 0.6–16.0 ± 0.2	Adolescents	Cross-sectional	Australia	Rugby league	Speed, change of direction speed, muscular power, maximal aerobic power
Gabbett & Seibold (2013) [15]	32	24 ± 3	Adults	Prospective cohort design	Australia	Rugby league	Strength, strength endurance, power, prolonged HIRA
Galvin et al. (2013) [29]	30	18.4 ± 1.5	Adolescents	Single-blind placebo controlled design	England	Rugby league & union	Repeated sprint training, speed, prolonged HIRA
Green et al. (2011) [6]	28	19 ± 1.3–19 ± 1.7	Adolescents	Cross-sectional	Ireland	Rugby union	Speed, change of direction ability
Hansen et al. (2011) [79]	40	23.7 ± 5.0	Adults	Cross-sectional	Australia	Rugby union	Speed, power
Holloway et al. (2008) [70]	12	21.5 ± 2.2	Adults	Cross-sectional	Australia	Rugby league	Anaerobic endurance
Jarvis et al. (2009) [10]	19	23.0 ± 5.4	Adults	Cross-sectional	Wales	Rugby union	Speed, agility, maximum aerobic power
Johnston & Gabbett (2011) [51]	12	22.7 ± 2.2	Adults	Randomized, counterbalanced cross over experimental	Scotland	Rugby league	Repeated sprint ability & effort
Johnston et al. (2015) [54]	31	16.5 ± 0.5	Adolescents	Between groups, repeated measures experimental design	Australia	Rugby league	HIRA
Johnston et al. (2015b) [60]	21	19.2 ± 0.7	Adolescents	Cross-sectional	Australia	Rugby league	HIRA, muscular strength, power
Kirkpatrick and Comfort (2013) [38]	24	18.7 ± 0.9	Adolescents	Cross-sectional	England	Rugby league	Power, strength, speed
Krause et al. (2015) [76]	485	U12-U15	Adolescent	Cross-sectional	Australia	Rugby union	Speed, power
Lombard et al. (2015) [7]	453	18.1 ± 0.7	Adolescents	Repeated cross-sectional design	South Africa	Rugby union	Strength, endurance, speed
Moore and Murphy (2003) [71]	15	22.5 ± 2.5	Adults	Cross-sectional	Australia	Rugby union	Anaerobic capacity
Meir et al. (2001) [58]	146	N/m	Adults	Cross-sectional	England and Australia	Rugby league	Speed, Muscle strength, power, endurance, agility
Parsonage et al. (2014) [39]	156	15 ± 7	Adolescents	Cross-sectional	UK	Rugby union	Power, speed, endurance capacity
Pienaar and Coetzee (2013) [28]	40	18.9 ± 0.4	Adolescents	Pre-posttest, randomized experimental design	South Africa	Rugby union	Power, acceleration, speed, agility, anaerobic capacity
Scott et al. (2015) [68]	55	15.6 ± 0.3–19.4 ± 0.5	Adolescents	Test retest, comparative cross-sectional	Australia	Rugby league	Prolonged HIRA
Serpell et al. (2010) [74]	30	≥ 18	Adolescents & Adults	Within subject & between subject experimental design	Australia	Rugby league	Agility
Smart and Gill (2013) [42]	44	15.3 ± 1.3	Adolescents	Pre-post experimental control design	New Zealand	Rugby union	Strength, power, speed, anaerobic and aerobic running
Smart et al. (2013) [52]	1161	*N/m	Adults	Retrospective, secondary data analysis	New Zealand	Rugby union	Strength, power, speed, repeated sprint ability.
Smart et al. (2014) [17]	510	*N/m	Adults	Retrospective, secondary data analyses	New Zealand	Rugby union	Strength, speed, power, repeated sprint ability
Till et al. (2016) [18]	81	U17-U19	Adolescents & Adults	Cross-sectional, Longitudinal	United Kingdom	Rugby League	Speed, Muscular power, strength Endurance,
Till et al. (2014a) [69]	133	15.5–20.1	Adolescents	Longitudinal	England	Rugby league	Power, speed, endurance, strength
Till et al. (2014b) [55]	75	13.0–19.9	Adolescents	Longitudinal	England	Rugby league	Power, speed, endurance, strength.
Till et al. (2015) [56]	130	U16-U20	Adolescents	Longitudinal	England	Rugby league	Power, speed, endurance, strength
Till and Jones (2015) [57]	121	12.8–15.5	Adolescents	Longitudinal	England	Rugby league	Power, speed, endurance
Till et al. (2011) [43]	1172	13.57 ± 0.27–15.57 ± 0.27	Adolescents	Longitudinal	United Kingdom	Rugby league	Muscular power, Speed, change of direction speed, maximal aerobic uptake
Till et al. (2013) [44]	81	13.6 ± 0.2	Adolescents	Longitudinal	United Kingdom	Rugby League	Muscular power, speed, change of direction, maximal aerobic power
Till et al. (2014c) [45]	81	13.62 ± 0.24	Adolescents	Longitudinal	United Kingdom	Rugby League	Muscular power, speed, change of direction speed, maximal aerobic power
Till et al. (2016b) [41]	580	U13-U15	Adolescents	Longitudinal	United Kingdom	Rugby League	Speed, Change of direction speed, Muscular power, maximal aerobic power
Till et al. (2013b) [46]	1172	U13-U15	Adolescents	Longitudinal	United Kingdom	Rugby League	Speed, muscular power, change of direction speed, maximal aerobic power
Till et al. (2016c) [66]	257	U15	Adolescents	Longitudinal	United Kingdom	Rugby league	Muscular power, speed, change of direction speed, maxiam aerobic power
Till et al. (2015b) [67]	580	13.60 ± 0.55–13.80 ± 0.72	Adolescents	Cross-sectional	United Kingdom	Rugby League	Speed, change of direction speed, muscular power, maximal oxygen uptake.
Till et al. (2010) [48]	683	13.6 ± 0.27–15.54 ± 0.27	Adolescents	Longitudinal	United Kingdom	Rugby league	Speed, change of direction speed, muscular power, maximal oxygen uptake.
Vaz et al. (2014) [12]	46	26.2 ± 2.8–26.7 ± 2.9	Adults	Cross-sectional	Portugal	Rugby union	Strength, speed, maximal aerobic power
Waldron et al. (2014a) [62]	28	15.1 ± 0.4–17.0 ± 0.4	Adolescents	Longitudinal	Australia	Rugby league	Speed, power, aerobic endurance
Waldron et al. (2014b) [63]	13	15.1 ± 0.3–17.0 ± 0.3	Adolescents	Longitudinal	Australia	Rugby league	Speed, power, aerobic endurance
Gabbett (2002b) [64]	66	24 ± 4	Adults	Cross-sectional	Australia	Rugby league	Power, speed, agility, maximal aerobic power
Gabbett (2006b) [40]	77	16.7–27.3	Adolescents & Adults	Cross-sectional	Australia	Rugby league	Speed, agility, maximal aerobic power

^a^high intensity running ability; ^b^ repeated high intensity exercise performance; ^ƪ^age was reported as mean ± standard deviation or range (for one sample of participants) or group range (if a study had more than two groups of participants); *N/m-not mentioned; Strength-denotes lower or upper body muscular strength; Power- denotes lower or upper body muscular power

### Physiological characteristics and the corresponding tests

Table 2 provides an overview of physiological characteristics, corresponding tests used to measure each construct in rugby and the absolute number of studies that used a specific physiological test. This review identified 15 physiological characteristics commonly evaluated among rugby players. These include speed, repeated-sprint and effort ability, repeated high-intensity exercise performance, prolonged high-intensity intermittent running ability/endurance, anaerobic endurance, maximal aerobic power and speed, agility, lower-body muscular power and strength, upper-body muscular strength and power, upper-body muscular endurance and abdominal endurance. However, there were no studies evaluating muscle flexibility of the rugby players that met the inclusion criteria.

**Table 2:** An overview of tests used to measure specific physiological characteristics as described in the included studies

Physiological construct*	Corresponding test(s)	Reference(s)	N
Speed	10 m, 20 m and 40 m sprint test	[30–41]	12
	10 m, 20 m, 30, and 60 m sprint test	[41–48]	8
	10 m and 40 m sprint test	[7, 10, 16, 27, 49, 61, 77]	7
	10 m and 20 m sprint test	[5, 18, 55–57, 69]	6
	5 m, 10 m and 20 m sprint test	[19, 29, 75]	3
	10 m, 20 m and 30 m sprint test	[17, 48, 52]	3
	10 m and 30 m sprint test	[6, 62]	2
	5 m, 10 m, 20 m and 40 m sprint test	[53, 59]	2
	10 m and 60 m sprint test	[66]	1
	10 m, 20 m, 30 m and 40 m sprint test	[64]	1
	10 m, 30 m and 40 m sprint test	[76]	1
	10 m, 20 m, 30 m, 40 m and 50 m sprint test	[8]	1
	5 m, 10 m and 30 m sprint test	[79]	1
	5 m and 10 m sprint test	[73]	1
	15 m and 40 m sprint test	[58]	1
	20 m sprint test	[63]	1
Repeated-sprint ability	Repeated 20 m sprint test	[16, 29, 49–51]	5
	Rugby specific repeated speed (RS^2^) test	[17, 52]	2
Repeated-effort ability	Repeated effort ability test	[51]	1
Repeated high intensity exercise performance	Repeated high intensity exercise (RHIE) Back test	[24]	1
	Repeated high intensity exercise (RHIE) RL Forward test	[24]	1
	Repeated high intensity exercise (RHIE) RU Forward test	[24]	1
Prolonged high-intensity intermittent running ability/Endurance	Yo-yo intermittent recovery test (level 1)	[15, 18, 53–56, 59, 60]	8
	Repeated 12 s sprint shuttle speed test	[16, 49, 50]	3
	Yo-yo intermittent recovery test (level 2)	[24]	1
	Multistage fitness test	[57]	1
	5 min run	[58]	1
Maximal aerobic power/uptake	Multistage fitness test	[7, 8, 10, 16, 27, 30–37, 40, 41, 43–46, 48–50, 61–67]	29
	Yo-yo intermittent recovery test (level 1)	[69]	1
	30–15 Intermittent Fitness test (30–15_IFT_)	[68]	1
	1500 m run (Metabolic Fitness Index)	[42]	1
Maximal aerobic speed/Anaerobic speed reserve	30–15 Intermittent Fitness test (30–15_IFT_)	[53, 59]	2
Anaerobic endurance	Triple 120 m shuttle (T120S) test	[70]	1
	Wingate 60 (w60) cycle test	[70]	1
	300 m shuttle run test	[71]	1
	400 m sprint test (Metabolic Fitness Index for Team Sports)	[42]	1
Change of direction speed/Agility	(Agility) 505 test	[16, 19, 36, 37, 41, 43–49, 53, 65–67, 72]	17
	L-run	[19, 31, 32, 34, 35, 40, 58]	7
	Illinois Agility test	[27, 30, 64]	3
	Modified 505 test	[19, 73]	2
	Change of direction speed test	[6, 74]	2
	Agility test	[75]	1
	Novel agility test (no specific name given)	[77]	1
Lower body muscular power	Vertical (Sargent) jump test	[15, 16, 30–36, 40, 49, 61, 64, 65, 73]	15
	Countermovement jump test (CMJ)	[18, 38, 39, 41, 43–48, 53, 55–57, 60, 62, 63, 66, 67, 69, 75, 76]	22
	Jump squat test	[13, 75, 77–79]	5
Lower body muscular strength	1 repetition maximum (RM) back squat	[5, 17, 18, 38, 55, 56, 69, 77, 80]	9
	1 RM box squat	[13, 42]	2
	3 RM back squat	[15, 60]	2
	Isometric squat on force plate	[75]	1
Upper body muscular power	2 kg medicine ball chest throw	[41, 43–48, 57, 66]	9
	20s push up test	[36]	1
	Overhead medicine ball throw	[73]	1
	Bench throw	[13]	1
	20s chin up test	[36]	1
	Plyometric Press-up	[60]	
Upper body muscular strength	1RM bench press	[5, 7, 17, 18, 27, 38, 42, 55, 56, 58, 69, 78, 80]	13
	1RM chin up test	[17, 42]	2
	3RM bench press	[15, 60]	2
	Push test	[27]	1
	Prone row	[18]	1
Upper body muscular endurance	60s push up test	[36]	1
	60s chin up test	[36]	1
	Bench press repetitions-to-fatigue at 60% 1RM	[81]	
	1RM Bench press repetitions-to-fatigue at 60 kg	[81]	1
	1RM Bench press repetitions-to-fatigue at 102.5 kg	[81]	
	Pull up test	[7]	1
	Body mass bench press with repetition	[15]	1
	30s plyometric push-up test	[58]	1
Abdominal endurance	60s sit-up	[58]	1

*RL* rugby league, *RU* Rugby union

*The physiological characteristic is written as described in the original article

The majority of these physiological characteristics had multiple tests for measurement. Overall, the 70 studies included in the review described 63 physiological tests: speed (8), upper-body muscular endurance (8), agility/change of direction speed (7), upper-body muscular power (6), upper-body muscular strength (5), prolonged high-intensity intermittent running ability/endurance (5), lower-body muscular strength (5), anaerobic endurance (4), maximal aerobic power (4), lower-body muscular power (3), repeated high-intensity exercise performance (3), repeated-sprint ability (2), repeated-effort ability (1), maximal aerobic speed (1) and abdominal endurance (1). Table 3 summarises the procedures for administering each physiological test identified.

**Table 3:** A descriptive summary of procedure for the tests identified as commonly used in the included studies

Physiological construct(s)	Tests identified	Basic description on how the tests were performed in included studies	Outcome measures	References
Speed	5 m, 10 m, 15 m, 20 m, 30, 40 m, 50 m and 60 m sprint tests	Players run along the 60 m distance from a pre-determined starting point. Running speed evaluated at 5 m, 10 m, 20 m, 30 m, 40 m, 50 m and 60 m using dual beam electronic timing gates.	Total sprint time per each distance (s)	[5–8, 10, 16–19, 27, 29–49, 52, 53, 55–67, 69, 73, 75–77, 79]
Repeated sprinting ability (RSA)	Repeated 20 m sprint tests	Players perform 10 or 12 maximal effort sprints over a 20 m distance with each sprint performed on a 20 or 30-s cycle. Recovery characterised by walking around the cone 10 m from the end of the sprint track.	Total repeated sprint time (s), percentage decrement, average heart rate (b.min^−1^), peak heart rate (b.min^−1^), rating of perceived exertion.	[16, 29, 49–51]
	Rugby-specific repeated speed (RS^2^) test	The test consists of three sets of three or four individual sprints performed maximally at set time intervals. Each set of sprints is separated by periods of standardised work where the players jog with a weighted bag. Players repeated sprints are measured using electronic timing gates over the same distance as speed (30 m for backs and 20 m for forwards and half backs).	Mean time per sprint (s), *fatigue, mean of 12 sprints for 20 m for forwards and the mean of 9 sprints for 30 m for backs	[17, 52]
Repeated effort ability (REA)	Repeated-effort test	The protocol comprises of 12 × 20 m sprints and tackles with each sprint commencing every 20s and the tackle performed after each 20 m sprint.	Total repeated effort time(s), % decrement, average heart rate (b.min^−1^), peak heart rate (b.min^−1^), rating of perceived exertion	[51]
Repeated high intensity exercise performance (RHIE)	RHIE Backs test	Each player complete 3 × 20 m sprints on a 20s cycle. After 3 sprints, players complete 2 tackles 10 m away with 20s recovery. This drill is repeated three times for each participant.	Individual sprint time (s), sum of sprint time (s), decrement in sprint time over the 3 sets of sprints (s)	[24]
	RHIE RL Forward test	Similar to the RHIE Backs test, except that players complete 5 tackles in each circuit.	Sum of sprint times (s), decrement in sprint time (s)	[24]
	RHIE RU Forward test	Each player complete 3 × 20 m sprints on a 20s cycle. After 3 sprints, players complete a ‘scrum sled shuttle’ four times. Then players repeat the sprint shuttles (3 × 20 m). After that, players tackle a tackle bag at 10 m four times	Total sprint time (s), decrement in sprint performance (s)	[24]
Prolonged high intensity intermittent running ability/Endurance	Yo-Yo intermittent recovery test (level 1)	Players perform 2 × 20 m runs back and forth at a progressively increasing speed keeping to a series of beeps/audio signals from compact disc. Players perform the test at level 1.	Total distance covered (m), last level reached	[15, 19, 53–56, 59, 60]
	Yo-Yo intermittent recovery test (level 2)	Same as above but the test is performed at level 2.	Total distance covered (m)	[24]
	Repeated 12 s sprint shuttle speed test	Players perform 8 × 12 s maximal effort shuttles (sprinting forward 20 m, turning 180 degrees and sprinting 20 m), each shuttle performed at 48 s cycle.	Total sprint distance, percentage decrement	[16, 49, 50]
	Multistage fitness test	Players run back and forth along a 20 m track keeping in time with the series of beeps on a compact disc with the speed progressively increased until volitional exhaustion.	Total distance covered (m)	[57]
	5 min run	Players are required to cover as much distance as possible around the course in a 5-min period.	Total distance covered (m)	[58]
Maximal aerobic fitness	Multistage(shuttle run) fitness test	Same as above	Number of shuttles/laps/levels completed, total distance covered (m), predicted VO_2MAX_	[7, 8, 10, 16, 27, 30–37, 40, 41, 43–46, 48–50, 61–67]
	Yo-yo intermittent recovery test (level 1)	Players perform 20 m runs back and forth at a progressively increasing speed keeping to a series of beeps/audio signals from compact disc. Players perform the test at level 1.	VO_2MAX_ predicted via the equation: distance run (m) × 0.0084 + 36.4	[69]
	30–15 Intermittent Fitness test (30–15_IFT_)	30s shuttle runs interspersed with 15 s periods of passive recovery. Players run back and forth between 2 lines 40 m apart at a pace governed by a pre-recorded beep.	Last stage reached, running velocity (V_IFT_)	[68]
	1500 m run (Metabolic Fitness Index for Team Sports)	Players would perform the 1500 m run on a synthetic running track.	Time taken to complete the distance (m)	[42]
Maximal aerobic speed/Anaerobic speed reserve	30–15 Intermittent Fitness test (30–15_IFT_)	30s shuttle runs interspersed with 15 s periods of passive recovery. Players run back and forth between 2 lines 40 m apart at a pace governed by a pre-recorded beep.	Maximal aerobic speed (MAS), Anaerobic speed reserve (ASR)	[53, 59]
Anaerobic endurance	Triple 120 m shuttle (T120S) test	Players perform 3 sets of 120 m shuttle sequences.	Time taken to complete the 120 m shuttle, maximum heart rate, blood lactate, rating of perceived exertion	[70]
	Wingate 60 (w60) cycle test	Each player will perform a 60s all out maximal effort on a cycle ergometer according to the Wingate protocol.	Maximal heart rate, blood lactate, rating of perceived exertion	[70]
	300 m shuttle run test	Players sprint maximally between two lines, 15 times, for a total distance of 300 m.	Total time to complete the run (s)	[51]
	400 m sprint test (Metabolic Fitness Index for Team Sports)	Players run maximally an entire lap of the track for 400 m.	Time to complete the run (s)	[42]
Agility/change of direction speed (CODS)	505 test	Players assume a starting position 10 m from timing gates. They accelerate as quickly as possible along the 15-m distance, pivot on the 5 m line or turn 180 degrees at the 15 m mark and return as quickly as possible through the timing gates placed 5 m from a designated turning point	Total time taken (s)	[16, 19, 36, 37, 41, 43–49, 53, 65–67, 72]
	L-run	Three cones placed 5 m apart in an ‘L’ shape. Players run as quickly as possible along the 5 m, turn left, run forward 5 m, turn 180 degrees and follow same course to finish and dual beam electronic timing gates used to record time.	Total time taken (s)	[19, 31, 32, 34, 35, 40, 58]
	Illinois Agility test	Players start lying in prone on the starting line. On a signal the players stand up and accelerate towards and around the cones set up. They can sprint for 9 m return to the starting line; they swerve in and out of the four cones completing two 9 m sprints to finish the agility course.	Total time taken to complete the course (s)	[27, 30, 64]
	Modified 505 test	Two timing gates placed 5 m apart from s designated turning point; unlike the traditional 505 test where players start at 10 m from the timing gates and therefore 15 m from the turning point, players start 5 m from the timing gates, pivot on the 5 m line and return as quickly as possible through the timing gates	Total time taken to complete the course (s)	[19, 73]
	Change of direction speed test	Players sprint forward 5 m then perform a 45 degree change of direction manoeuvre to pass through either left or right finish gate.	Total time taken to complete the course (s)	[6, 74]
	Agility test	Players sprint 5 m through the first timing gates to the second timing gates and sprint back to the third timing gate positioned at the starting line 5 m from the first and sprint back to the fourth timing gate positioned 5 m away from the second time to finish the course	Total time taken to complete the course (s)	[75]
	Novel agility test (no specific name given)	Players sprint 1 m at a 45 degree angle, turn around a marker cone, sprint at 45 degrees for 10 m back to starting line. Here they make 135 degree turn around another cone and sprint 20 m in a straight line perpendicular to the goal line	Total time taken to complete the course (s)	[77]
Lower body muscular power	Vertical jump test	Using a Yardstick device or a board, players stand with feet flat on the ground, fully extended arms and hands, and mark the standing reach height. After assuming a crouch position, players spring upward and touch the yardstick device or the board at the highest possible point.	Vertical jump height calculated as the distance from the highest point reached during and the highest reaching during the vertical jump	[15, 16, 30–36, 40, 42, 49, 61, 64, 65, 73]
	Countermovement jump test (CMJ)	Players put hands on hips and jump from the jump mat or portable force plate from a standing position moving from a self-selected depth in squatting and jump explosively as far as possible. A Takei vertical jump metre may be used.	Jump height, peak power, vertical power was estimated by equation: CMJ power (W) =61.9 × Jump height + 36.0 × body mass-1822.	[18, 38, 39, 41, 43–48, 53, 55–57, 60, 62, 63, 66, 67, 69, 75, 76]
	Jump squat test	Players self-select foot position and lower the Olympic bar 40 kg to a self-selected depth and then the players are required to jump as explosively as possible. The bar will be resting on upper trapezius. Loaded jump squat may have a resistance of 20 kg to 100kgs conducted using the Plyometric Power System (PPS) or 40 kg jump squat from a force plate.	Mechanical power output	[13, 75, 77–79]
Lower body muscular strength	One repetition maximum back squat (1RM BS)	Using an Olympic bar and free weights, players back squat until the top of the thigh is parallel with the ground and return to a standing position to record one repetition maximum.	Maximum weight lifted (kgs)	[5, 17, 18, 38, 55, 56, 69, 77, 80]
	Isometric squat on force plate	Players stand on a force plate with the bar of a Smith Machine resting on upper trapezius at a height which results in an angle of 135 degrees knee flexion.	Peak force generated (n)	[75]
	1 RM box squat	Players use a self-selected foot position and lower themselves to sitting position briefly on the box and then return to standing position	One repetition maximum (kgs)	[13, 42]
	3RM full squat exercise	Players perform this with the free weight Olympic-style barbell. Players lower their body until thighs are past parallel with the floor and fully extend the hip and knee joints	Maximum weight lifted (kgs)	[15]
Upper body muscular strength	One repetition maximum bench press (1RM BP)	Players in supine, feet flat on floor, hips and shoulders in contact with the bench, lower the bar to touch the chest and push the bar until the elbows are locked out.	Maximum weight lifted (kg)	[5, 7, 17, 27, 38, 42, 55, 56, 58, 69, 78, 80]
	3RM bench press	The test is performed as above at three repetition maximum	Maximum weight lifted (kg)	[15, 60]
	1RM chin up test	Players use a reverse underhand grip (palms facing towards face). Players instructed to start from a stationary position with arms fully extended and complete a repetition with the chin moving over the bar	One repetition maximum (kgs)	[17, 42]
	Push-Up test	Players begin in prone, with hands on the floor, thumbs shoulder width apart and elbows fully extended. Players are instructed to descend to the tester fist placed on the floor below the players’ sternum and then ascend until the elbows are straight.	The number of push-ups in one minute (n)	[27]
	1RM Prone row	Participants lay face down on a bench with the bench height determined by the players reach when the arms are fully extended. Participants have to pull the barbell towards the bench and the lift will be recorded if both sides of the barbell touch the bench	Maximum weight lifted (kg)	[18]
Upper body muscular power	20s push up test	Players assume prone position, body lowered until the elbows are 90 degrees, followed by a return to the starting position with arms fully extended.	Time taken to complete 20 full push ups (s)	[36]
	20s chin up test	Players assume a hanging position on the bar, hands shoulder width apart with supinated grip and arms extended. Players are to raise the body until the chin touched the top of the bar with the head in neutral position.	Maximum number of chin-ups in 20 s	[36]
	Overhead ball throw test	Players stand with 1 ft aligned with the a line marked on the ground facing the throwing direction, with a 3 kg medicine ball held in both hands behind the head, each player is required to plant the front foot with the toe behind the line and to throw the medicine ball overhead as far as possible.	Maximum distance thrown (m)	[73]
	Chest throw test	Players throw a 2 kg medicine ball horizontally as far as possible while seated with the back against the wall	Maximum distance thrown (m)	[41, 43–48, 57, 66]
	Bench throw test	Players use a self-selected hand position and lower the bar to a self-selected depth approximately 90 degrees at the elbow and then throw or propel the bar vertically as explosively as possible.	Maximum weight thrown (kgs)	[13]
Upper body muscular endurance	60s push up test	Players assume prone position, body lowered until the elbows are 90 degrees, followed by a return to the starting position with arms fully extended.	Maximum number of push-ups in 60s	[36]
	60s chin up test	Players assume a hanging position on the bar, hands shoulder width apart with supinated grip and arms extended. Players are to raise the body until the chin touched the top of the bar with the head in neutral position.	Maximum number of chin ups in 60s	[36]
	Bench Press repetitions-to-fatigue (BP RTF)	Players perform bench press repetitions as possible till fatigue at two markedly different resistances of 60-kgs and 102.5-kgs	Number of repetitions (n)	[81]
	Bench press repetitions-to-fatigue at 60% 1RM	Players perform bench press repetitions as possible till fatigue with a resistance of 60% of their one repetition maximum bench press	Number of repetitions at 60% 1RM BP	[81]
	Pull up test	Using an underhand grip, and the hands 10–15 cm apart, players start in the hanging position and ascended to a position with the chin above the bar and then return to starting position with arms extended.	Maximal number of completed pull-ups	[7]
	Body mass bench press with repetition	Using players body mass as resistance for as many repetitions as possible until fatigue	Number of repetitions (n)	[15]
	30s Plyometric push up	Participants would take a push-up position supporting self on the palm of left or right hand with the other hand placed on the top of a 5 kg medicine ball. The players then lower themselves to the ground until elbows are 90 degrees; they then forcefully pushes back with complete extension of the arms, while shifting the hand on the ground across to the new position on the medicine ball. Similarly, the hand on the ball shift across to a position approximately 2 shoulder widths on the opposite side of the ball	Maximum number of repetitions in designated time period	[58]
Abdominal endurance	60s Sit up	Participants would sit with feet flat on the floor and held in position by another player. The arms would be crossed at the shoulders and knees bent at an angle approximately 90 degrees. On command, the players would curl the trunk so that elbows touch the front of the thighs and then return to starting position	Maximum number of repetitions in 60s	[58]

VO_2MAX_- maximal aerobic power estimated using regression equations; s = seconds; *calculated as a percent change in sprint time predicted from the linearized change derived from all sprints performed; b.min^−1^ = beats per minute; RL = Rugby League; RU = Rugby Union; m = meters; vVo_2_max = velocity at maximal oxygen uptake also known as MAS (maximal aerobic speed); ASR = Anaerobic speed reserve calculated as the difference between individual maximum velocity (maxV) and MAS; N = newton; n = number of repetitions; kgs = Kilograms; 1RM bench press-one repetition maximum bench press

#### Speed

Running speed was the most common physiological characteristic evaluated among rugby players. Of the 70 studies, 51 (72.9%) examined the speed characteristics of rugby players. Straight-line sprinting was commonly measured over eight distances of 5 m, 10 m, 15 m, 20 m, 30 m, 40 m, 50 m and 60 m recorded using dual beam electronic timing gates (Tables 2 and 3). Of the 50 studies, 98% assessed the speed of rugby players over multiple distances. Twelve (24%) studies specifically used multiple linear distances of 10 m, 20 m and 40 m [30–41] and eight (16%) used the 10 m, 20 m, 30 m and 60 m sprint tests for the speed evaluation of rugby players [41–48].

#### Repeated sprint and effort ability

There were seven (10.0%) studies that evaluated repeated-sprint abilities of rugby players. However, only two tests were commonly used in these studies to evaluate the construct. The Repeated 20 m Sprint test was used in five of the seven studies [16, 29, 49–51]. The test involves players performing 10 or 12 maximal effort sprints over a 20 m distance with each sprint performed on a 20 or 30s cycle [16, 29, 49–51]. In addition, there were two studies that evaluated the repeated sprint abilities of rugby participants using the Rugby-Specific Repeated Speed (RS^2^) test [17, 52]. The Repeated-Effort Ability test was used in one study to investigate the physiological characteristic of repeated-effort ability in rugby players [51]. The protocol comprises of 12 × 20 m sprints and tackles with each sprint commencing every 20s and the tackle performed after each 20 m sprint [51].

#### Repeated high-intensity exercise performance

The ability to perform repeated high-intensity exercises by rugby players was assessed using specifically developed Repeated High-Intensity Exercise (RHIE) tests. Three tests were used in a study by Austin et al. [24] and were modified for RU backline players, RU forward players and RL forward players.

#### Prolonged high-intensity intermittent running ability/endurance

Fourteen (20.0%) studies investigated the measurement of a physiological characteristic termed “prolonged high-intensity intermittent running ability” or endurance [15, 16, 18, 24, 49, 50, 53–60]. Of the 14 included studies, eight used the Yo-Yo Intermittent Recovery Level 1 (Yo-Yo IRT1) test [15, 18, 53–56, 59, 60] and three utilised the Repeated-12 s Sprint Shuttle Speed test [15, 49, 50]. The Yo-Yo IRT1 involves performing 2 × 20 m runs back and forth at a progressively increasing speed keeping to a series of beeps/audio signals from compact disc [15, 53, 54]. The Repeated 12 s Sprint Shuttle speed test involves players performing 8 × 12 s maximal effort shuttles (sprinting forward 20 m, turning 180 degrees and sprinting 20 m) and each shuttle is performed at 48 s cycle [16, 49, 50]. In addition, there was only one study that evaluated the construct of “prolonged high-intensity intermittent running ability” using the Yo-Yo Intermittent Recovery Level 2 (Yo-Yo IRT2) test [24].

#### Maximal aerobic power and speed

Of the 70 studies, 32 (45.7%) studies estimated the maximal aerobic power of rugby players. Of these studies, 29 (90.6%) used the Multistage Fitness test [7, 8, 10, 16, 27, 30–37, 40, 41, 43–46, 48–50, 61–67]. Other tests used in singular studies to estimate maximal aerobic power included the 30–15 Intermittent Fitness test (30–15_IFT_) [68], 1500 m run [42] and the Yo-Yo IRT1 [69]. Maximal aerobic speed was evaluated using the 30–15 Intermittent Fitness test (31-15_IFT_) [53, 59]. The test involves performing 30s shuttle runs conducted at a pace governed by a pre-recorded beep and interspersed with 15 s periods of passive recovery. The test begins at 8 km/h and increased to 0.5 km/h at each successive running shuttle [53].

#### Anaerobic endurance

Three (4.28%) studies assessed the anaerobic endurance of rugby players. One study compared results of rugby players on two tests of anaerobic endurance: Triple 120 m (T120S) test and the Wingate 60 (w60) cycle test [70]. Other tests used in singular studies included the 300 m Shuttle Run test [71] and the 400 m Sprint test [42].

#### Change of direction speed/agility

The change of direction speed/agility of rugby players was commonly measured in a number of studies. It was the third most commonly measured physiological characteristic in the included studies. In total, 33 (47.1%) studies examined the change of direction speed or agility of rugby players. Of these studies, 17 (51.5%) used the 505 test [16, 19, 36, 37, 41, 43–49, 53, 65–67, 72] and seven (21.2%) used the L-run test [19, 31, 32, 34, 35, 40, 58]. The 505 test involves players assuming a starting position 10 m from timing gates and accelerate as quickly as possible along the 15-m distance, pivot on the 5 m line or turn 180 degrees at the 15 m mark and return as quickly as possible through the timing gates placed 5 m from a designated turning point [16, 19, 36, 37, 49, 53, 72]. On the other hand, the L run involves three cones placed 5 m apart in an ‘L’ shape and players have to run as quickly as possible along the 5 m, turn left, run forward 5 m, turn 180 degrees and follow same course to finish [19, 31, 32, 34, 35, 40]. Other tests used in the included studies are the Illinois Agility test (*n* = 3) [27, 30, 64], Modified 505 test (*n* = 2) [19, 73] and Change of Direction Speed test (CODS) (*n* = 2) [6, 74].

#### Lower-body muscular power and strength

Lower-body muscular power was the second most commonly investigated physiological characteristic in rugby participants. Of the 70 studies, 42 (60.0%) studies included in this review examined that construct. Of these studies, 15 (35.7%) used the Vertical Jump (VJ) test [15, 16, 30–36, 40, 42, 49, 61, 64, 65, 73]. The VJ involves using a Yardstick device or a board and players are instructed to stand with feet flat on the ground, fully extended arms and hands, and mark the standing reach height. After assuming a crouch position, players are requested to spring upward and touch the yardstick device or the board at the highest possible point [15, 16, 30–36, 40, 42, 49, 61, 64, 65, 73]. Twenty-two (52.4%) studies used the Countermovement Jump (CMJ) test [18, 38, 39, 41, 43–48, 53, 55–57, 59, 60, 62, 63, 66, 67, 69, 75, 76]. The difference in the two vertical jump tests is that the CMJ involves participants standing with their hands positioned on the hips and usually jump from a jump mat as high as possible [18]. The Jump Squat (JS) test was used in five studies [13, 75, 77–79].

Of the 70 studies, 14 (20.0%) assessed lower-body muscular strength of rugby players. The most frequently used test was the One Repetition Maximum Back Squat (1RM BS). The test was used in nine of the fourteen studies [5, 17, 18, 38, 55, 56, 69, 77, 80]. Using an Olympic bar or free weights, players are instructed to back squat until the top of the thigh is parallel with the ground and return to a standing position to record 1RM [5, 17, 38, 55, 56, 69, 77, 80]. In addition, two studies used the 1RM Box Squat [13, 42] and 3RM Back Squat [15, 60], respectively.

#### Upper-body muscular power and strength

Nineteen (27.1%) studies evaluated the upper-body muscular strength of rugby players. Of these studies, 13 (68.4%) used the 1RM Bench Press [5, 7, 17, 18, 27, 38, 42, 55, 56, 58, 69, 78, 80]. The 1RM BP test involves players in supine, feet flat on floor, hips and shoulders in contact with the bench. The players are instructed to lower the bar to touch the chest and push the bars until the elbows are locked out, recording the 1RM [5, 7, 17, 27, 38, 42, 55, 56, 69, 78, 80]. Two studies used the 1RM Chin-Up test [17, 42] and the 3RM Bench Press [15, 60]. On the other hand, there were 12 (17.1%) studies that examined that upper-body muscular power for rugby players. The frequently used test in the included studies was the 2 kg Medicine Ball Chest Throw [41, 43–48, 57, 66]. Other tests used in singular studies included the 20s Push-Up and 20s Chin-Up tests [36], Overhead Medicine Ball Throw test [73], Bench Throw test [13].

#### Upper-body and abdominal muscular endurance

Of the included studies, upper body muscular endurance was assessed in five studies only (7.14%). One singular study utilised two tests: 60s Push-Up and Chin-Up tests [36]. Another study used the 1RM Bench Press Repetitions-to-Fatigue test at 60 kg, 102.5 kg and at 60% of 1RM [81]. Other tests used in singular studies included the Pull-Up test [7] and the body mass Bench Press with repetition test [15] and the 30s Plyometric push-up test [58]. Abdominal endurance was identified in one study and was assessed using the 60s Sit-Up test [58].

## Stage 2: Methods

Stage 1 allowed us to identify tests commonly used for the measurement of physiological characteristics of speed, repeated sprint ability and effort, repeated high-intensity exercise performance, prolonged high-intensity intermittent running ability/endurance, maximal aerobic power and speed, anaerobic endurance, change of direction speed/agility, lower and upper –body muscular strength, power, and abdominal endurance. Briefly, the second stage of the systematic review was conducted to provide evidence on the measurement properties of each identified physiological test from Stage 1. The ultimate aim, however, was to identify one physiological test per physiological construct with the strongest level of evidence on measurement properties on best evidence synthesis.

### Literature search, search strategy and eligibility criteria

The electronic databases used for literature search in Stage 1 were used for Stage 2. Initially, we searched specifically for full-text studies with the primary purpose of investigating the measurement properties (reliability, validity and responsiveness) of the previously identified physiological tests in male rugby participants. This was done for the determination of physiological tests validated in the population of interest to the researcher (MC) for his future studies using rugby participants [21, 82]. However, provided that there was no satisfactory information found on the measurement properties for certain physiological tests in rugby studies, it was pre-planned that we would search for the evidence from clinimetric studies on related, intermittent, collision team sports such as Australian Rules football (AFL), American football, Gaelic football and Soccer. But, included studies from related sports had to have a similar description of the procedure of the test as described in rugby-related studies. In cases where there were major adjustments according to the researcher (MC) in the procedure of test between sports such studies were excluded. A search strategy proposed by Terwee et al. [83] guided the selection of keywords (see Additional file 2). The strategy for searching clinimetric studies in rugby and related sports consisted of a combination of following search themes (i, ii, iii, iv) and (i, ii, iv, v), respectively, connected with the Boolean term AND:

i. Test-specific terms: Vertical jump test OR Yo-Yo intermittent recovery test OR repeated 20 m sprint test.
ii. Measurement property-related terms: Psychometric* OR measurement* OR clinimetric*.
iii. Rugby-related terms: rugby OR rugby union OR rugby league.
iv. Target population-related search terms: adult OR adolescent OR male
v. Other team sport-related terms: Australian Rules football OR American football OR Soccer.

### Data extraction

The selection process of the identified articles was conducted as described previously in stage 1. Subsequently, data extraction was conducted using two independent people (SO and TM). All the data extracted was put on Microsoft Excel and was given to two other independent assessors (JD and TM) for further verification purposes on the accuracy of the data. The following data were extracted: publication details (first author, year of publication), title, purpose of the study, age of the participants, country, sport context, physiological construct evaluated, test(s) used to measure the construct, and the measurement properties assessed (reliability, validity and responsiveness). For the measurement properties, the following data were extracted: type of reliability or validity, interval period for test-retest and inter-rater studies, sample size and the results obtained for each physiological test.

### Quality assessment of the clinimetric studies and measurement properties

The Consensus-based Standards for the Selection of health Measurement Instruments (COSMIN) checklist was used to evaluate the methodological quality of the included studies. Briefly, the COSMIN evaluates nine measurement property items (internal consistency, reliability, measurement error, content validity, construct validity (i.e. structural validity, hypothesis testing, cross-cultural validity), criterion validity and responsiveness) (Table 4). It also provides standardised information for evaluating the quality of each item based on design requirements and statistical methods [84, 85]. The COSMIN scoring system per measurement property is based on a point rating scale (poor to excellent) and the overall rating for the methodological quality of each study is obtained by taking the lowest score [83, 84].

**Table 4:** Rating of the Quality of the statistical outcomes to determine measurement properties

Measurement property	Definition	(Rating) Quality criteria^a, b^
Reliability		
Internal consistency	The extent to which items in a (sub)scale are intercorrelated, thus measuring the same construct	(+) Factor analyses performed on adequate sample size (7 * # items and >100) AND Cronbach’s alpha(s) calculated per dimension AND Cronbach’s alpha(s) between 0.70 and 0.95; (?) No factor analysis OR doubtful design or method (−) Cronbach’s alpha(s) 0.70 or O0.95, despite adequate design and method. (0) No information found on internal consistency.
Reproducibility		
Agreement	The extent to which the scores on repeated measures are close to each other (absolute measurement error)	(+) MIC < SDC OR MIC outside the LOA OR convincing arguments that agreement is acceptable. (?) Doubtful design or method OR (MIC not defined AND no convincing arguments that agreement is acceptable) (−) MIC > SDC OR MIC equals or inside LOA, despite adequate design and method; (0) No information found on agreement.
Reliability	The extent to which patients can be distinguished from each other, despite measurement errors (relative measurement error)	(+) ICC > 0.70 OR k > 0.70 (?) Doubtful design or method (e.g., time interval not mentioned) (−) ICC or weighted Kappa ≤0.70, despite adequate design and method (0) No information on reliability found
Validity		
Content Validity	The extent to which the domain of interest is comprehensively sampled by the items in the questionnaire	(+) A clear description is provided of the measurement aim, the target population, the concepts that are being measured, and the item selection AND target population and (investigators OR experts) were involved in item selection; (?) A clear description of above-mentioned aspects is lacking OR only target population involved OR doubtful design or method; (−) No target population involvement; (0) No information found on target population involvement.
Construct validity	The extent to which scores on a particular questionnaire relate to other measures in a manner that is consistent with theoretically derived hypotheses concerning the concepts that are being measured	(+) Specific hypotheses were formulated AND at least 75% of the results are in accordance with these hypotheses; (?)Doubtful design or method (e.g., no hypotheses); (−) Less than 75% of hypotheses were confirmed, despite adequate design and methods; (0) No information found on construct validity.
Criterion validity (predictive or concurrent	The extent to which scores on a particular questionnaire relate to a gold standard	^C^(+) correlation with standard ≥0.70 OR no statistically significant differences between the two tests found OR sensitivity and specificity ≥0.70 OR convincing arguments that gold standard is “gold” AND correlation with gold standard >0.70; (?)No convincing arguments that gold standard is “gold” OR doubtful design or method; (−) Correlation with standard <0.70 or AUC < 0.70 OR statistically significant differences between outcome measures and gold standard OR sensitivity or specificity <0.70
Responsiveness	The ability of a questionnaire to detect clinically important changes over time	(+) SDC or SDC < MIC OR MIC outside the LOA OR RR O 1.96 OR AUC > 0.70; (?) Doubtful design or method; (−) SDC or SDC > MIC OR MIC equals or inside LOA OR RR < 1.96 OR AUC < 0.70, despite adequate design and methods. (0)No information found on responsiveness.
Floor and ceiling effects	The number of respondents who achieved the lowest or highest possible score	(+) ≤ 15% of the respondents achieved the highest or lowest possible score (?) Doubtful design or method (−) > 15% achieved the highest and lowest possible score despite adequate designs and methods (0) No information found on interpretation
Interpretability	The degree to which one can assign qualitative meaning to quantitative scores	(+) Mean and SD scores presented of at least four relevant subgroups of patients and MIC defined; (?) Doubtful design or method OR less than four subgroups OR no MIC defined; (0) No information found on interpretation.

*MIC* minimal important change, *SDC* smallest detectable change, *LOA* limits of agreement, *ICC* Intraclass correlation, *SD* standard deviation

^a^(+) positive rating; (?) indeterminate rating; (−) negative rating; (0) no information available

^b^Doubtful design or method = lacking of a clear description of the design or methods of the study, or any important methodological weakness in the design or execution of the study

Two reviewers (JD and TM) with prior COSMIN experience evaluated the methodological quality of each study included in Stage 2. It was pre-planned that disagreements were resolved by discussion with the third person (CT) until a consensus was reached. In addition to the methodological quality assessment with the COSMIN, the quality criteria for rating of measurement properties checklist as given by Terwee et al. [86] was used to rate each measurement property in the included articles as ‘positive’, ‘negative’ or ‘questionable’ depending on the results of the property reported (Table 4). Studies with “poor” methodological qualities were not analysed for the quality of the results on the measurement properties.

### Best evidence synthesis: levels of evidence

To help synthesise results from numerous studies on the same physiological construct, the “best evidence synthesis” was performed by the primary author (MC). The best evidence synthesis rating was determined based on the number of studies that have investigated the measurement property, the overall COSMIN score, and the rating and consistency of the measurement property result (positive, indeterminate, and negative) [87]. The possible levels of evidence are “strong” (when consistent findings in multiple studies of good methodological quality were found or in one excellent methodological quality study), “moderate” (when consistent findings in multiple studies of fair methodological quality were found or in one study of good methodological study), “limited” (if only one study of fair methodological quality was found), “conflicting” (conflicting findings) and “unknown” (if only studies of poor methodological quality were found or no studies) [87].

## Results: Stage 2

### Characteristics of included studies

**Fig. 2:**
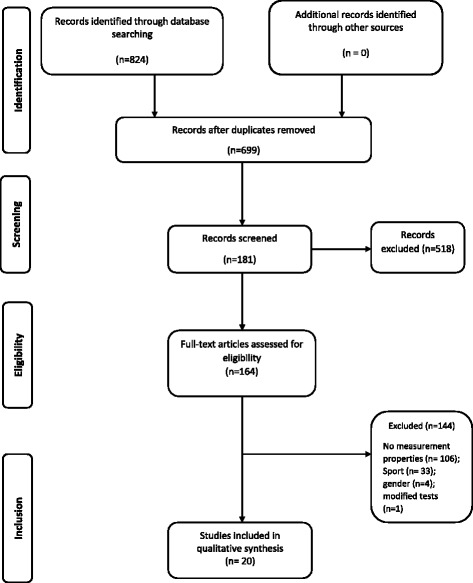
Flow chart for the search and selection of stage 2 articles

Figure 2 shows a flow chart for the selection of the studies. Of 824 studies identified from the electronic databases, 20 met the inclusion criteria. The majority of the studies did not meet the inclusion criteria because they did not report on measurement properties. The general characteristics of the included studies and a summary of the measurement properties evaluated in each study are summarised in Table 5. The studies were conducted in Australia (*n* = 9), Denmark, Brazil, Belgium (*n* = 2), Norway, Ireland, Iran, Italy and Croatia (*n* = 1). The age of the participants in the included studies ranged from 12 to 36 years.

**Table 5:** Characteristics of included studies from stage 2 and the psychometric properties assessed

Authors	Title	Purpose of the study	Age	Country	Sport	Test(s)	Construct measured	Properties evaluated
Austin et al. (2013) [24]	Reliability and sensitivity of a repeated high- intensity exercise performance test for Rugby league and Rugby Union	To examine the reliability and sensitivity of 3 repeated high-intensity exercise tests (RHIE)	24 ± 4 (Backs); 24 ± 3 (RU forwards); 24 ± 2 (RL forwards)	Australia	RL and RU	RHIE Backs test RHIE RL Forward test RHIE RU Forward test	Repeated high-intensity exercise	Reliability
Baker (2009) [81]	Ability and validity of 3 different methods of assessing upper-body strength-endurance to distinguish playing rank in professional rugby league players	To compare the ability and validity of 3 different methods of assessing strength-endurance	Study 1 = 20.0 ± 1.2–24.9 ± 3.0 years Study 2 = 19.5 ± 1.7–25.0 ± 3.3 years	Australia	RL	BP RTF 60% 1RM BP RTF 60 kg BP RTF 102.5 kg	Upper-body strength-endurance	Validity
Duthie et al. (2006) [99]	The reliability of ten-meter sprint time using different starting techniques	To compare the reliability of 10 m sprint times when using different starting techniques	17 ± 0.7 years	Australia	RU	10 m sprint test with foot start 10 m sprint test with standing start 10 m sprint test with thumb start	Speed	Reliability
Gabbett et al. (2008) [19]	Speed, change of direction, and reactive agility of Rugby League players	To investigate the discriminative ability of speed, change of direction speed, and reactive agility tests	23.6 ± 5.3 years	Australia	RL	5 m sprint test 10 m sprint test 505 test Modified 505 test Lrun test	Speed, Agility	Reliability, Validity
Green et al. (2011) [6]	A valid field test protocol of linear speed and agility in Rugby Union	To investigate the reliability and construct validity of a field test protocol	19 ± 1.67–19 ± 1.30 years	Ireland	RU	10 m sprint test 30 m sprint test Change of direction speed	Speed, Agility	Reliability, Validity
Holloway et al. (2008) [70]	The Tripple-120 m shuttle test: A sport-specific test for assessing anaerobic fitness in Rugby League Players	To design a sport specific test for anaerobic endurance and compare the validity of the test with the Wingate 60-s cycle test	21.5 ± 2.15 years	Australia	RL	Tripple-120 m shuttle test	Anaerobic endurance	Validity
Johnston and Gabbett (2011) [51]	Repeated-sprint and effort ability in Rugby League players	To assess the test-retest reliability of repeated sprint and repeated effort tests	22.7 ± 2.2 years	Australia	RL	Repeated ability sprint test Repeated effort test	Repeated sprint ability and effort	Reliability
Serpell et al. (2010) [74]	The development of a new test of agility for Rugby League.	To develop a reliable and valid agility test	>18 years	Australia	RL	Change of direction speed test	Agility	Reliability, Validity
Scott et al. (2015) [68]	Reliability and usefulness of the 30–15 Intermittent fitness test in Rugby League	Examined the reliability and usefulness of the 30 Intermittent Fitness test	15.6 ± 0.3–19.4 ± 0.5 years	Australia	RL	30–15 Intermittent fitness test	Intermittent running ability	Reliability
Ingebrigtsen et al. (2012) [97]	Yo-Yo IR2 testing of elite and sub-elite soccer players: Performance, heart rate response and correlations to other interval tests	To correlate the Yo-Yo Intermittent recovery test level 2 with other frequently used tests in elite soccer	20 ± 3–26 ± 7 years	Denmark and Norway	Soccer	Yo-Yo intermittent recovery test (level 2)	Prolonged high-intensity intermittent running ability	Validity
Deprez et al. (2014) [88]	Reliability and validity of the Yo-yo intermittent recovery test (level 1) in young soccer players	To investigate the test-retest reliability and construct validity from the Yo-Yo Intermittent recovery test level 1	12.5 ± 0.6–16.2 ± 0.6 years	Belgium	Soccer	Yo-Yo intermittent recovery test (level 1)	Prolonged high-intensity intermittent running ability	Reliability, Validity
Krustrup et al. (2003) [89]	The Yo-yo intermittent recovery test: Physiological response, reliability and validity	To examine the reproducibility and validity of the Yo-Yo intermittent recovery test level 1	Range: 25–36 years	Denmark	Soccer	Yo-yo intermittent recovery test (level 1)	Prolonged high-intensity intermittent running ability	Reliability, Validity
Krustrup et al. (2006) [98]	The Yo-Yo IR2 test: Physiological response, reliability and application to elite soccer	To examine the physiological response and reliability of the Yo-Yo intermittent recovery test level 2	Range: 22–30 years	Denmark	Soccer	Yo-yo intermittent recovery test (level 2)	Prolonged high-intensity intermittent running ability	Reliability
Markovic & Mikulic (2011) [93]	Discriminative ability of the Yo-yo intermittent recovery test (level 1) in prospective young soccer players	To evaluate the discriminative ability of the Yo-yo intermittent recovery test level 1	12.0–18.9 years	Croatia	Soccer	Yo-yo intermittent recovery test (level 1)	Prolonged high-intensity intermittent running ability	Validity
Fanchini et al. (2014) [94]	Are the Yo-yo intermittent recovery test levels 1 and 2 both useful? Reliability, responsiveness and interchangeability in young soccer players	To compare the reliability, internal responsiveness and interchangeability of the Yo-Yo intermittent recovery test level 1	17 ± 1 years	Italy	Soccer	Yo-yo intermittent recovery test (level 1) Yo-yo intermittent recovery test (level 2)	Prolonged high-intensity intermittent running ability	Reliability, Validity Responsiveness
Buchheit & Rabbani (2014) [95]	The 30–15 Intermittent fitness test versus the Yo-yo intermittent recovery test level 1: relationship and sensitivity to training.	To examine the relationship between Yo-Yo intermittent recovery test and the 30–15 Intermittent Fitness test and compare the sensitivity of both tests to training	15.4 ± 0.5 years	Iran	Soccer	Yo-yo intermittent recovery test (level 1)	Prolonged high-intensity intermittent running ability	Validity, Responsiveness
Deprez et al. (2015) [96]	The Yo-Yo intermittent recovery test level 1 is reliable in young high-level soccer players	To investigate the test-retest reliability of the Yo-yo intermittent recovery test level 1	13.9 ± 0.5–18.1 ± 0.4 years	Belgium	Soccer	Yo-yo intermittent recovery test level 1	Prolonged high-intensity intermittent running ability	Reliability
Da Silva et al. (2011) [91]	Yo-Yo IR2 and Margaria test: Validity, reliability and maximum heart rate in young soccer players	To evaluate the reliability, construct validity of the Yo-Yo intermittent recovery test and of the Margaria test.	14 ± 0.8 years	Brazil	Soccer	Yo-Yo intermittent recovery test (level 2)	Prolonged high-intensity intermittent running ability	Reliability, Validity
De Salles et al. (2012) [90]	Validity and reproducibility of the Sargent jump test in the assessment of explosive strength in soccer players	To check the validity, inter and intra-evaluators reproducibility of the Sergeant jump test.	14.3 ± 0.66 years	Brazil	Soccer	Sargent (vertical jump) jump test	Lower-body muscular power	Reliability, Validity
Veale et al. (2010) [92]	The Yo-yo intermittent recovery test (level 1) to discriminate elite junior Australian football players	To evaluate the discriminative validity of the Yo-yo intermittent recovery test	16.6 ± 0.5 years	Australia	Australian football	Yo-Yo intermittent recovery test (level 1)	Prolonged high-intensity intermittent running ability	Validity

RU = Rugby union; RL = Rugby League; Bench Press repetition-to-fatigue with resistance at 60% 1RM = BP RTF 60% 1RM; Bench Press repetition-to-fatigue with resistance at 60 kg and 102.5 kg = BP RTF 60 kg and BP RTF 102.5 kg

Out of the 63 tests identified in stage 1, 20 studies described the measurement properties of only 21 tests. The tests were the 5 m, 10 m, 20 m and 30 m Speed tests (speed), 20 m Repeated-Sprint test (repeated sprinting ability), Repeated-Effort test (repeated effort ability), three Repeated High-Intensity Exercise tests (repeated high-intensity exercise performance), Yo-Yo IRT1 and 2 (prolonged high-intensity running ability), T120 s (anaerobic endurance), 505 test (agility), Modified 505 test (agility), L run (agility), Change of Direction Speed test (agility), Sergeant Jump test (lower-body muscular power), and three Bench Press Repetition-to-Fatigue tests (upper-body strength-endurance).

Of the 21 tests, 18 were studied for their measurement properties in rugby. The Yo-Yo Intermittent Recovery Level 1 and 2 and the Sergeant Jump tests had their measurement properties derived from other related sports (Soccer and Australian Rules football). Other than the tests mentioned above, there was no evidence on the measurement properties either in rugby or related sports for all the other tests identified in stage 1. However, for the 21 tests identified in stage 2, none of the tests had all the measurement properties investigated. But, the majority of the studies (*n* = 7) investigated the reliability and validity of one or more physiological tests [6, 19, 74, 88–91].

### Measurement properties and methodological quality assessments

Tables 6 and 7 provide an overview of the measurement properties for the identified physiological tests and the COSMIN rating of methodological quality for the studies per measurement property. Table 8 shows rating of the quality of the results on the measurement properties based on the quality rating criteria of measurement properties checklist given by Terwee et al. [86]. The results on the measurement properties for the physiological tests derived from studies of “poor” methodological quality were excluded from the rating.

**Table 6:** Measurement properties (reliability and measurement error) of the physiological tests and methodological quality scores

Test	Reliability (Intra-rater, inter-rater, test-retest) and measurement error			COSMIN
	Design (interval period)	*n*	Results	Score
RHIE Backs test [24]	Test-retest (2 days)	12	Total sprint time, ICC = 0.82 (CV = 0.1–3.2%); Percentage decrement, ICC = 0.78 (CV = 4.2–49.5%)	Poor
RHIE RL Forward test [24]	Test-retest (2 days)	12	Total sprint time, ICC = 0.97 (CV = 0.1–4.9%); Percentage decrement, ICC = 0.86 (CV = 1.4–48.2%)	Poor
RHIE RU Forward test [24]	Test-retest (2 days)	12	Total sprint time, ICC = 0.94 (CV = 0.1–5.1%); Percentage decrement, ICC = 0.88 (CV = 0.6–35.8%)	Poor
5 m sprint [19]	Test-retest (2 days)	42	Fastest time, ICC = 0.84 (% TE = 3.2)	Fair
10 m sprint [19]	Test-retest (2 days)	42	Fastest time, ICC = 0.87 (%TE = 1.9)	Fair
10 m sprint with foot start [99]	Test-retest (7 days)	15	ICC = 0.86 (TE% = 0.9)	Poor
10 m sprint with standing start [99]	Test-retest (7 days)	15	ICC = 0.92 (TE% = 0.88)	
10 m sprint with thumb start	Test-retest (7 days)	15	ICC = 0.92 (TE% = 1.00)	
10 m sprint [6]	Test-retest (3 days)	11	Average sprint time, ICC = 0.88 (SEM = 0.08)	Poor
20 m sprint [19]	Test-retest (2 days)	42	Fastest time, ICC = 0.96 (% TE = 1.3)	Fair
30 m sprint [6]	Test-retest (3 days)	11	Average sprint time, ICC = 0.97 (SEM = 0.06)	Poor
505 test [19]	Test-retest (2 days)	42	Fastest time, ICC = 0.90 (%TE = 1.9)	Fair
Modified 505 test [19]	Test-retest (2 days)	42	Fastest time, ICC = 0.92 (%TE = 2.5)	Fair
L run test [19]	Test-retest (2 days)	42	Fastest time, ICC = 0.95 (%TE = 2.8)	Fair
CODS test [6]	Test-retest (3 days)	11	Average time, ICC = 0.87 (SEM = 0.06)	Poor
CODS test [74]	Test-retest (7 days)	15	Average time, ICC = 0.87 (SEM = 0.01)	Poor
T120S test [70]	Test-retest (4 days)	12	Total time taken, *r =* 0.74 (*p* = 0.006)	Poor
20 m RSA test [51]	Test-retest (7 days)	12	Total sprint time, ICC = 0.96 (%TE = 1.5)	Poor
			Decrement (%), ICC = 0.91 (%TE = 22.5)	
			Average heart rate, ICC = 0.56 (%TE = 3.5)	
			Peak heart rate, ICC = 0.88 (%TE = 1.4)	
			Rating of perceived exertion, ICC = 0.78 (%TE = 5.5)	
REA test [51]	Test-retest (7 days)	12	Total time, ICC = 0.82 (%TE = 2.3)	Poor
			Decrement (%), ICC = 0.91 (%TE = 6.7	
			Average heart rate, ICC = 0.96 (%TE = 0.9)	
			Peak heart rate, ICC = 0.88 (%TE = 1.5)	
			Rating of perceived exertion, ICC = 0.59 (%TE = 3.3)	
30–15_IFT_ test [68]	Test-retest (9 days)	55	Maximal intermittent running velocity (V_IFT_), ICC = 0.89 (CV% = 1.9); SWC = 0.21	Good
		13	Heart rate, ICC = 0.96 (CV% = 0.6); SWC = 1 beats per minute	Poor
Yo-Yo IR1 [88]	Test-retest (8 days)	35	Under 13: Total distance, ICC = 0.82 (CV% = 17.3); LoA = 0.98 ×/÷ 1.27, range = 0.77–1.24	Poor
		32	Under 15: Total distance, ICC = 0.85 (CV% = 16.7); LoA = 0.89 ×/÷1.30, range = 0.68–1.16	
		11	Under 17: Total distance, ICC = 0.94 (CV% = 7.9); LoA = 0.94 ×/÷ 1.15, range = 0.82–1.08	
Yo-Yo IR1 [89]	Test-retest (within 1 week)	13	Total distance, *r* = 0.98 (CV% = 4.9)	Poor
Yo-Yo IR1 [94]	Test-retest (7 days)	24	Total distance, ICC = 0.78 (CV = 7.3%)	Poor
Yo-Yo IR2 [94]	Test-retest (7 days)	24	Total distance, ICC = 0.93 (CV = 7.1%)	Poor
Yo-Yo IR1 [96]	Test-retest (3 measurements within 1 week intervals)	22	Under 15: Total distance, ICC = 0.92 (CV% = 6.8–7.5); 95% ratio LoA (test 1 vs. test 2) =1.17 */÷ 1.24; 95% ratio LoA (test 2 vs. 3) = 0.96 */÷ 1.23; 95% ratio limit (test 1 vs. 3) = 1.13 */÷ 1.28.	Poor
		10	Under 17: Total distance, ICC = 0.95 (CV% = 3.1–5.4); 95% ratio LOA (test 1 vs. test 2) = 1.09 */÷ 1.13; 95% ratio LoA (test 2 vs. 3) = 0.97 */÷ 1.09; 95% ratio LoA (test 1 vs. 3) = 1.06 */÷ 1.15.	
		4	Under 19: Total distance, ICC = 0.87 (CV% = 3.0–6.9); 95% ratio LoA (test 1 vs. test 2) = 1.02 */÷ 1.11; 95% ratio LoA (test 2 vs. 3) = 0.88 */÷ 1.12; 95% ratio LoA (test 1 vs 3) = 0.90 */÷ 1.22.	
Yo-Yo IR2 [98]	Test-retest (2 days)	29	Total distance, CV% = 9.6%.	Poor
Yo-Yo IR2 [91]	Test-retest (7 days)	18	Total distance, ICC = 0.38 (CV% = 11)	Poor
Vertical (Sargent) jump test [90]	Intra-rater (testing sessions separated by 2 h)	45	ICC = 0.99 (95% CI = 0.99–1.00)	Fair
	Inter-rater	45	ICC = 1.00 (95% CI = 0.99–1.00)	Fair

Sign diff = significant differences; b/w = between; CV% = Coefficient of Variation expressed as a percentage; CI = confidence interval; ICC = Intraclass correlation coefficient; r = Pearson correlation coefficient; * highest effect size calculated between groups; SWC = smallest worthwhile change;; IFT = Intermittent fitness test; TE% = Percent typical error of measurement; CODS = Change of direction speed; T120S = Tripple-120 m shuttle test; r = Pearson’s product moment correlations; RSA = repeated sprint ability; REA = repeated effort ability; SWC = smallest worthwhile change; 95% ratio LoA = limits of agreement; Yo-Yo IR1 and 2 = Yo-Yo intermittent recovery tests 1 and 2

**Table 7:** Measurement properties (validity and responsiveness) of the physiological tests and methodological quality scores

Test	Validity			COSMIN	Responsiveness			COSMIN
	Type	*n*	Results	Score	Design	*n*	Results	Score
BP RTF 60 [81]	Hypothesis testing (Known group validity)	38	Sign diff b/w groups NRL (36.1 ± 7.2) vs. SRL (28.0 ± 5.6)	Fair	–	–	–	–
BP RTF 102.5 [81]	Hypothesis testing (Known group validity)	38	Sign diff b/w groups NRL (12.5 ± 4.3) vs. SRL (5.9 ± 3.9)	Fair	–	–	–	–
BP RTF 60% 1RM [81]	Hypothesis testing (Known group validity)	26	No sign diff b/w NRL and SRL players	Poor	–	–	–	–
5 m sprint test [19]	Hypothesis testing (Known group validity)	42	Sign diff b/w groups (First grade RL players vs. Second grade RL players) Effect Size = 0.68	Fair	–	–	–	–
10 m sprint test [19]	Hypothesis testing (Known group validity)	42	Sign diff b/w groups (First grade RL players vs. second grade RL players) Effect size = 0.85	Fair	–	–	–	–
10 m sprint test [6]	Hypothesis testing (Known group validity)	28	Sign diff b/w (Club RU players vs. Academy RU players) Effect size = 2.86	Poor	–	–	–	–
30 m sprint test [6]	Hypothesis testing (Known group validity)	28	Sign diff b/w (club RU players vs. Academy RU players) Effect size = 1.61	Poor	–	–	–	–
505 test [19]	Hypothesis testing (Known group validity)	42	No sign diff b/w between groups Effect size = 0.28	Fair	–	–	–	–
Modified 505 test [19]	Hypothesis testing (Known group validity)	42	No sign diff b/w groups Effect size = 0.32	Fair	–	–	–	–
L run [19]	Hypothesis testing (Known group validity)	42	No sign diff b/w groups Effect size = 0.28	Fair	–	–	–	–
CODS test [6]	Hypothesis testing (Known group validity)	28	Sign diff b/w groups. Effect size = 2.23	Poor	–	–	–	–
CODS test [74]	Hypothesis testing (Known group validity)	30	No sign diff b/w groups (Low performance group, *n* = 15 vs. High performance group, n = 15)	Poor	–	–	–	–
T120S test [70]	Criterion validity	12	Sign corr in maximum heart rate b/w the 2 trials of T120S and W60 cycle test (*r* = 0.63 and 0.71). No sign corr b/w 2 trials of T120S and W60 cycle test for post 3 min lactate (*r* = 0.11 and 0.10).	Poor	–	–	–	–
Yo-Yo IR2 [97]	Hypothesis testing (Known group validity)	51	Sign diff b/w elite vs. sub-elite soccer players.	Poor	–	–	–	–
	Hypothesis testing (convergent validity)	12 39	Sign corr b/w Yo-Yo IR2 and Yo-Yo IR1 (*r* = 0.74, *p* < 0.01) for the elite players. Sign corr b/w Yo-Yo IR2 and Yo-Yo IR1 (*r* = 0.76, *p* < 0.01) for sub-elite players.	Poor				
	Hypothesis testing (convergent validity)	12 39	Sign corr b/w Yo-Yo IR2 and 35 m repeated sprint ability test (*r* = −0.74, *p* < 0.01) for elite players. Moderate corr observed for sub-elite (*r* = −0.34, *p* < 0.05)	Poor				
	Criterion validity	13 12	Moderate corr for sub-elite players b/w Yo-Yo IR2 and treadmill test (*r* = 0.48, *p* < 0.01). No significant corr for the elite players (*r* = 0.59, *p* < 0.10)	Poor				
Yo-Yo IR1 [97]	Hypothesis testing (Known group validity)	51	Sign diff b/w elite (*n* = 12) vs. sub-elite (*n* = 39) soccer players	Poor	–	–	–	–
	Hypothesis testing (convergent validity)	12 39	Very large corr b/w Yo-Yo IR1 and 35 m repeated sprint time (*r* = −0.80, *p* < 0.01) for elite players (n = 12). Large corr b/w Yo-Yo IR1 and 35 m repeated sprint time (*r* = −0.51, *p* < 0.05) for sub-elite players (n = 39)	Poor				
	Criterion validity	12 39	Very large corr. b/w Yo-Yo IR1 and VO_2MAX_ for elite players (r = 0.76, *p* < 0.01). Very large corr b/w Yo-Yo IR1 and VO_2MAX_ for sub-elite players (*r* = 0.73, *p* < 0.01).	Poor				
Yo-yo IRT1 [92]	Hypotheses testing (Known group validity)	60	Sign diff b/w groups (*P* < 0.001). *ES = 3.78 elite Australian rules football (n = 20) vs. healthy group (*n* = 20).	Poor	–	–	–	–
Yo-yo IRT1 [88]	Hypotheses testing (Known group validity)	208	Sign diff b/w groups (p < 0.001) ES = 0.94 (90% CI = 0.46–1.43) b/w U15 Elite vs. Sub-elite	Poor	–	–	–	–
Yo-yo IRT1 [89]	Hypotheses testing (Convergent validity)	22	Sign corr b/w Yo-yo test performances and fitness performances during soccer match assessed using time motion analysis (*r* = 0.53–0.71, p < 0.05)	Poor	Repeated measures, 4 testing sessions [pre-preparation, mid preparation, start season, end season]	10	Sign diff in Yo-yo mean distance covered between preseason measures and seasonal measures (*p* < 0.05) Sign diff in heart rate measures b/w preseason and seasonal measures (*p* < 0.05)	Poor
	Criterion validity	17	Sign corr b/w Yo-yo test performances and time to fatigue (*r* = 0.79, p < 0.05) and maximal oxygen uptake (*r* = 0.71, *p* < 0.05)					
Yo-Yo IRT1 [93]	Hypotheses testing (Known group validity)	106	Sign group differences in YY IRT1 among age categories (F = 25.3; *p* < 0.001). *ES = 4.17 (U 13 vs. U 19) p < 0.01	Poor	–	–	–	–
Yo-Yo IRT1 [94]	Hypotheses testing (Convergent validity)	24	Sign corr b/w Yo-Yo IRT1 and Yo-Yo IRT2 (*r* = 0.56–0.84)	–	Repeated measures [(3 testing sessions of Yo-yo IRT1 before 11 wks of training + matches and 2 testing sessions post training + matches]	24	ES = 0.9 (90%CI = 0.66–1.18); SWC = 3.7%; MDC = 20.2%; % changes after training = 14.5%; Probability of substantial changes btwn pre-and post-measures = 99.9%	Poor
Yo-Yo IRT2 [94]	Hypotheses testing (Convergent validity)	24	Sign corr b/w Yo-Yo IRT1 and Yo-Yo IRT2 (r = 0.56–0.84).	poor	Repeated measures [(3 testing sessions of Yo-yo IRT2 before 11 wks of training + matches and 2 testing sessions post training + matches]	24	ES = 0.4 (90%CI = 0.17–0.69); SWC = 4.8%; MDC = 19.5%;	
Yo-Yo IR1 [95]	Hypotheses testing (Convergent validity)	14	Large corr b/n Yo-yo IRT1 and 30–15 IFT (*r* = 0.75, 90%CI = 0.57–0.86)	Poor	Pre and post measures interspaced by an 8-week training intervention	14	Within-test % changes = +35% (90% CI = 24–45) for Yo-yo IRT1 vs. +7% (90% CI = 4–10) for 30–15 IFT ES for the changes (standardised differences): Yo-yo IRT1 = 1.2 vs. 1.1 for 30–15 IFT	Poor
Yo-Yo IRT2 [98]	Criterion validity	13	A sign corr b/w Yo-yo IR2 and time to fatigue in the incremental running test (r = 0.74, p < 0.05)	Poor				
Yo-Yo IRT2 [91]	Hypotheses testing (Concurrent validity)	18	High positive corr found b/w Yo-Yo IRT2 and PRT >85% MHR during the match (*r* = 0.71, p = 0.001)	Poor	–	–	–	
Vertical (Sargent) jump test [90]	Criterion validity	45	ICC = 0.99 (95% CI = 0.97–1.00) *p* = 0.001	Fair	–		–	–

*PRT* performance of time of remaining above 85% MHR in the game, *Yo-Yo IR1 and 2* Yo-Yo intermittent recovery test level 1 and 2, *T120S* Triple 120 m shuttle run test, *CODS* Change of direction speed test, *ES* effect size, *SWC* smallest worthwhile change, *MDC* minimal detectable change, *30–15 IFT* 30–15 Intermittent fitness test, *BP RTF* bench press repetitions to fatigue test

**Table 8:** Overall quality score by study and rating of measurement properties for the physiological tests

Test	Reliability			Construct validity		Criterion	Responsiveness	Interpretability
	Intra	Inter	Test- retest	Known group	Convergent/Concurrent			
BP RTF 60 [81]	0	0	0	+	0	0	0	0
BP RTF 102.5 [81]	0	0	0	+	0	0	0	0
30–15_IFT_ [68]	0	0	+	0	0	0	0	0
5 m sprint test [19]	0	0	+	+	0	0	0	0
10 m sprint test [19]	0	0	+	+	0	0	0	0
20 m sprint test [19]	0	0	+	+	0	0	0	0
505 test [19]	0	0	+	–	0	0	0	0
Modified 505 test [19]	0	0	+	–	0	0	0	0
Lrun test [19]	0	0	+	–	0	0	0	0
Sargent (vertical) jump test [90]	+	+	0	0	0	?	0	0

? = doubtful design or method; 0 = no information; + = positive rating; − = negative rating; criterion = criterion validity

#### Yo-Yo intermittent recovery level 1 (Yo-Yo IR1) test

Of the 20 studies included in the review, seven investigated at least one measurement property of the Yo-Yo IR1 test (Table 5). Validity was the most commonly studied measurement property with six studies evaluating at least one type of validity [88, 89, 92–95]. There was evidence on known-group validity [88, 92, 93], convergent [89, 94, 95] and criterion validity [89] of the Yo-Yo IR1 test. However, all the six studies were rated “poor” on methodological quality mainly because of the inadequate sample sizes used in the validity analysis. Reliability was the second most commonly studied measurement property with four studies evaluating test-rest reliability (Table 5) [88, 89, 94, 96]. The test-retest intervals ranged from within one week to eight days [88, 89, 94, 96]. On methodological quality, all the studies investigating the reliability of the Yo-Yo IR1 were rated “poor”. In all these studies, the sample size had the lowest score and therefore determined the total score for the study. Another measurement property investigated for the Yo-Yo IR1 was responsiveness. However, responsiveness of the Yo-Yo IR1 test was reported in two studies of “poor” methodological quality [94, 95].

#### Yo-Yo intermittent recovery level 2 (Yo-Yo IR2) test

Of the 20 studies included in the review, four studies provided evidence on at least one measurement property of the Yo-Yo IR2 test (Table 5) [91, 94, 97, 98]. Validity and reliability were the most commonly studied measurement properties of the test [91, 94, 97, 98]. Three studies evaluated the test-retest reliability of the Yo-Yo IR2 with a seven day interval between the assessments [91, 94, 98]. However, all the three studies were rated “poor” on methodological quality mainly because of small sample sizes used for the reliability analysis. On the other hand, there were four studies that investigated the validity of the Yo-Yo IR2 test (Table 5) [91, 94, 97, 98]. Two studies provided evidence on convergent [94, 97] and criterion [97, 98] validity of the Yo-Yo IR2 test. In addition, singular studies investigated the known-group validity [97] and concurrent validity of the test [91]. All the studies were, however, rated “poor” on methodological quality. Responsiveness of the Yo-Yo IR2 test was examined in one study of “poor” methodological quality [94].

#### Speed tests

##### 5 m sprint test

Only one “fair” study investigated the measurement properties (reliability and validity) of the 5 m sprint test (Table 5) [19]. The 5 m sprint test was found to have positive rating [i.e. Intraclass Correlation Coefficient (ICC) > 0.70] for the test-retest reliability (Tables 6 and 8) [19]. The same study provided evidence on the construct validity of the test (Table 7). A positive rating for the known-group validity was found for the 5 m sprint test as specific hypotheses were formulated and at least 75% of the results were in accordance with these hypotheses (Table 8). There was no evidence on the responsiveness found for the test.

##### 10 m sprint test

Three different studies investigated the measurement properties of the 10 m sprint test (Table 5) [6, 19, 55]. Reliability was the most commonly studied measurement property. All the three studies had test-retest reliability evidence for the 10 m sprint test, with an interval of two to seven days between the assessments [6, 19, 99]. However, two of the studies were rated “poor” on methodological quality [6, 99]. In one “fair” study, a positive rating for the test-retest reliability (ICC = 0.87) of the 10 m sprint test was found [19]. Validity of the 10 m sprint test was assessed in two studies [6, 19]. The most common type of validity studied was construct validity (known-group validity). One study was rated as “poor” on methodological quality [6]. In that study, a positive rating of construct validity was found for the 10 m sprint test. There was no evidence found on the responsiveness of the test.

##### 20 m sprint test

Only one “fair” study investigated the measurement properties (reliability and validity) of the 20 m sprint test (Table 5) [19]. The 20 m sprint test was found to have positive rating for the test-retest reliability (Tables 6 and 8) [19]. The same study provided evidence on the construct validity of the test (Table 7). A positive rating for the known-group validity was found for the 20 m sprint test as specific hypotheses were formulated and at least 75% of the results were in accordance with these hypotheses (Table 8). There was no evidence on the responsiveness for the test.

##### 30 m sprint test

Test-retest reliability evidence of the 30 m sprint test was provided by one study rated “poor” on methodological quality [6]. The study used a sample size of 11 participants to establish the reliability of the test with three days between the test-retest assessments. In the same study, the 30 m sprint test was also assessed for its known-group validity [6]. However, the study was also rated “poor” on quality for the construct validity. There was no evidence found on the responsiveness of the test.

#### Repeated-sprint ability (RSA) test

One study assessed the test-retest reliability of repeated sprint ability test with assessments being conducted after seven days (Tables 5 and 6) [51]. The study was rated of “poor” methodological quality mainly because of small sample size used in the reliability analysis. There was no evidence on validity or responsiveness found for the test.

#### Repeated-effort ability (REA) test

One study assessed the test-retest reliability of repeated-effort ability test with assessments being conducted after seven days [51]. The study was rated of “poor” methodological quality mainly because of small sample size used in the reliability analysis. There was no evidence on validity found for the test.

#### Repeated high-intensity exercise (RHIE) tests

One study evaluated the test-retest reliability of three different repeated high-intensity exercise tests, namely, the repeated high-intensity exercise backs test, repeated high-intensity exercise rugby union forward test, and the repeated high-intensity exercise rugby league forward test [24]. The quality of the study was, however, rated “poor” mainly because of the small sample size per reliability analysis utilised for each test. There was no information on the validity or responsiveness of any of these tests in the literature.

#### 30–15 intermittent fitness test (30–15 _IFT_)

One study assessed the test-retest reliability of the 30–15 Intermittent Fitness test with nine days separating the two assessments [68]. For the measure of reliability for the primary outcome of maximal intermittent running velocity (V_IFT_), the study was rated as of “good” methodological quality. A positive rating (ICC = 0.89) for the test-retest reliability was reported for the test. Validity of the test was assessed in one study (Tables 5 and 7) [95]. The study was, however, rated “poor” on quality for the convergent validity of the 30–15 Intermittent Fitness test [95].

#### Triple 120-m shuttle test (T120S)

One study examined the test-retest reliability of the Triple 120 m shuttle test for anaerobic endurance using a four day interval between assessments [70]. On the other hand, the same study evaluated the criterion validity of the test against the Wingate 60s (W60) cycle test. The study used a small sample size of 12 rugby league players both for the reliability and the validity study and was rated “poor” on methodological quality. No information was found on the responsiveness of the test.

#### Agility/change of direction speed tests

##### 505 test

One study examined both test-retest reliability (over two days) and the construct validity of the 505 test [19]. The study was rated “fair” on methodological quality and a positive rating (ICC = 0.90) was reported for the test-retest reliability. For the construct validity, a negative rating was found for the 505 test as the results of the test showed an unexpected marginal effect size (ES = 0.28) because there were no significant difference between groups on the performance of the test. No information on responsiveness was found for the test.

##### Modified 505 test

Reliability of the Modified 505 test was investigated in one study [19]. The study was “fair” on methodological quality because of the large sample size. A positive rating (ICC = 0.92) on the test-retest reliability was found for the test. The same study investigated the construct validity of the test. The study had “fair” methodological quality on validity. A negative rating of construct validity (known-group validity) was found for the Modified 505 test as there was no significant difference between groups (ES = 0.32). Therefore, less than 75% of the results were in accordance with the hypotheses. No information was found for the responsiveness of the test.

##### L run test

One study examined both the test-retest reliability (over two days) and the construct validity of the L run [19]. The study was rated “fair” on methodological quality and a positive rating (ICC = 0.95) was reported for the test-retest reliability. For the construct validity, a negative rating was found for the L- run test as the results of the test showed an unexpected marginal effect size (ES = 0.28). There was no information found on responsiveness of the test.

##### Change of direction speed test

Two studies reported on the reliability of the change of direction speed test [6, 74]. The test-retest interval ranged between three to seven days. The same studies provided evidence on the construct validity (known-group validity) of the test [6, 74]. However, the two studies were rated “poor” on methodological quality for both reliability and validity. There was no information found on responsiveness of the test.

#### Sergeant (vertical) jump test

For the Sargent Jump test, there was only one study which was found evaluating inter and intra-rater reliability of the test [90]. Intra-rater reliability was assessed with testing sessions separated by two hours whilst inter-rater reliability assessments were separated by two days. The study was rated “fair” on methodological quality. A positive rating for intra-reliability (ICC = 0.99) and inter-rater reliability (ICC = 1.00) was reported for the test. The same study evaluated the validity of the Sergeant Jump test and showed positive criterion validity against the Jump Platform (JP) test using 45 soccer participants. The study was rated “fair” quality for criterion validity. There was no information found on responsiveness of the test.

#### Bench press repetitions-to-fatigue tests

One study examined the construct validity of three different upper-body strength-endurance tests, namely, bench press repetitions-to-fatigue at 60% of one repetition maximum test (BP RTF 60% 1RM), bench press repetitions-to-fatigue at 60 kg (BP RTF 60) and bench press repetitions-to-fatigue at 102.5 kg (BP RTF 102.5) [81]. For the BP RTF 60 and 102.5, the study was rated “fair” on methodological quality because of the adequate sample size (*n* = 38). A positive rating of construct validity was found for the two tests. However, for the construct validity of the BP RTF 60% 1RM test, the study was rated “poor”. There was no information on the reliability or responsiveness of the three tests in measuring upper body strength-endurance.

### Best evidence synthesis: level of evidence

A summary of best evidence synthesis are presented in Table 9. The synthesis was derived from information on the rating of the methodological qualities of the studies and results on the measurement properties of the tests. Only studies with “fair” to “good” methodological quality were used to determine the level of evidence per test for each studied measurement property. Best evidence synthesis showed moderate evidence to support the test-retest reliability of the 30–15_IFT_ test. Limited evidence was found to support the test-retest reliability and the known-group validity of the 5 m sprint test, 10 m speed test, 20 m speed test, 505 test, modified 505 test and the Lrun tests. There is also limited level of evidence for inter/intra-rater reliability and criterion validity of the Sergeant (vertical) jump test. Furthermore, there was limited evidence on the known group validity of the upper-body strength endurance tests of Bench-Press repetitions-to-fatigue at 60 and 102.5 kgs. There is unknown evidence available on the measurement properties of all the other tests identified in stage 1.

**Table 9:** Best level synthesis for the physiological tests

Test	Reliability			Hypothesis testing			
	Inter	Intra	Test-retest	Known group	Convergent	Criterion	Responsiveness
5 m sprint test [19]	0	0	+	+	0	0	0
10 m sprint test [19]	0	0	+	+	0	0	0
20 m sprint test [19]	0	0	+	+	0	0	0
505 test [19]	0	0	+	0	0	0	0
Modified 505 [19]	0	0	+	0	0	0	0
Lrun [19]	0	0	+	0	0	0	0
Sargent jump test [90]	+	+	0	0	0	+	0
BP RTF 60 [81]	0	0	0	+	0	0	0
BP RTF 102.5 [81]	0	0	0	+	0	0	0
30–15_IFT_ [68]	0	0	++	0	0	0	0

+/− = limited evidence (One study of fair methodological quality); ++/−− moderate evidence (consistent findings in multiple studies of fair methodological quality OR in one study of good methodological quality; 0 = no evidence or information available. All the other tests had unknown level of evidence on measurement properties because of poor methodological quality

## Discussion

The aim of the present systematic review was two-fold. Firstly, we systematically reviewed 70 studies in Stage 1 to identify physiological characteristics evaluated in rugby and the corresponding tests used to measure each construct. Thereafter, 20 studies were systematically reviewed in Stage 2 to provide an overview on the measurement properties of the physiological tests identified in the studies. Most of the included studies from stage 1 were from Australia, United Kingdom, New Zealand, and South Africa. This probably reflects the popularity of the sport of rugby in these respective countries. The fact that there were an almost equal number of adult and adolescent rugby studies indicates that rugby is extensively studied in junior and senior players. It is also possible to speculate that the sport is equally popular among junior and senior players.

One most important finding that emerged from stage 1 was that there are a number of physiological characteristics that are commonly investigated among rugby players. Fifteen physiological characteristics were identified. This extensiveness probably confirms wide interest researchers have in physiological characteristics. The interest could be linked with suggestions that success in rugby is highly dependent on physiological characteristics [75]. With increased professionalism and competition, there has been extensive investment in research towards establishing physical qualities important for successful performance in professional rugby. Moreover, this breadth of physiological characteristics under investigation potentially highlights the physical nature of the sport and diversity in attributes needed to meet the physical demands of the game. It is well-established that rugby is a physical sport requiring participants to partake in challenging physical collisions such as scrummaging, tackling, aggressive mauling and rucking which require optimal muscular strength, power and endurance [5]. This gives rationale to the preponderance of studies investigating lower and upper body muscular power [15, 16, 30–36, 40, 49, 61, 64, 73], lower and upper body muscular strength [5, 7, 18, 27, 38, 42, 55, 56, 69, 78, 80] and muscular endurance [7, 15, 36, 81]. In addition, rugby players variably cover 5000 to 7000 m during match play and engage intermittently in high-intensity efforts which require exceptional agility, anaerobic and aerobic capacity, speed, repeated sprinting and effort ability and generation of high levels of concentric and eccentric force production [53, 75]. This also provides justification for numerous studies investigating attributes such as speed, agility, prolonged high-intensity intermittent running ability, repeated sprint ability and explosive lower leg power [7, 16, 19, 30–38, 40, 49, 51, 53, 70, 72, 76].

Stage 1 findings also showed that almost all physiological characteristics had multiple tests for measurement. For example, this review showed that change of direction speed/agility is often evaluated using the 505, modified 505, Illinois Agility test, change of direction speed test among other tests. However, it was surprising to discover that for all the tests identified in Stage 1, none had all the measurement properties (reliability, validity and responsiveness) investigated using rugby participants. In addition, of the 63 tests identified in Stage 1, only 21 had information on at least one of the measurement properties from rugby and related sports. This suggests that there is limited reporting of the measurement properties for tests commonly used in rugby in the literature. This was particularly evident for the property of responsiveness. All these findings are interesting and raise questions on the rationale for selection of tests by researchers in the field of rugby. For example, speed was the most commonly studied physiological characteristic in the included studies. It was frequently measured from linear distances varying between 5 m and 60 m (Table 2). The commonly tested sprinting distances for speed were, however, the 10 m, 20 m and 40 m. Professional rugby studies have provided the evidence that players seldom sprint distances greater than 40 m in a single bout [100]. This probably justifies the predominance use of the 10 m, 20 m and 40 m sprint tests in assessing rugby players in the literature [30–40]. In addition, straight-line sprinting is reported to be broken down into three phases: acceleration, attainment of maximal speed, and maintenance of maximal speed [101]. This is also possibly justifies the use of more than one sprinting distance for assessing speed as all these distinct qualities of speed should be evaluated separately. Although there could be plenty of reasons researchers prefer a specific test over others, literature generally recommends the use of feasible, reliable, valid and responsive tests [102]. This review found that there is dearth of high-quality studies (according to the COSMIN scoring system) investigating the measurement properties of speed tests using rugby participants. Best evidence synthesis only showed that there is limited evidence for the test-retest reliability and the known-group validity of the 5 m sprint test, 10 m sprint test and the 20 m speed test.

Repeated-sprint ability has also been reported to be extremely important in rugby given the high-intense and intermittent nature of the sport [100]. This review showed that the construct is commonly measured using the Repeated 20 m sprint test and the Rugby-Specific Repeated Speed test. There were no high-quality studies found investigating the measurement properties of these tests in rugby. Only one study of “poor” methodological quality was found evaluating the test-retest reliability of the repeated 20 m sprint test using 12 rugby participants [51]. One needs to apply caution when adopting or using these tests in future studies using rugby players. High-quality future studies may need to explore the measurement properties of these tests. Repeated-sprint ability tests have been reported to underestimate the repeated high-intensity exercise demands of rugby [24]. To overcome the shortcomings of the repeated 20 m sprint test, Austin et al. [24] assessed the reliability of three repeated high-intensity exercise tests specifically developed for backline players, RU forward players and RL forward players. The study was, however, rated as of “poor” methodological quality because of the small sample size per reliability analysis of each test and short interval (2 days) for the test-retest assessments.

There is dearth of high-quality studies investigating the measurement properties of the Yo-Yo intermittent recovery (Level 1 and 2) tests in rugby. This is despite the popularity of the tests in assessing prolonged high-intensity intermittent running ability/endurance and maximal aerobic power among rugby players [15, 24, 53–56, 69]. This creates a need for future studies to specifically evaluate the measurement properties of the test using rugby participants. However, much of the information on measurement properties of these tests reported in rugby studies is referenced from validation studies conducted using participants from other sports. There are multiple studies providing the evidence of the measurement properties (reliability, validity and responsiveness) of the tests in other related intermittent sports such as Soccer and Australian Rules football [88, 89, 91–98]. However, no high-quality studies were found evaluating the measurement properties of the test according to the COSMIN guidelines. All the studies included in this review assessing the measurement properties of the tests showed “poor” methodological quality. The major drawbacks in all these studies were mainly related to the issues of inadequate sample sizes and lack of a clear description of the expected hypotheses. There were also no studies evaluating the measurement properties of other tests of prolonged high-intensity intermittent running ability such as the repeated 12 s sprint shuttle speed tests.

There were four tests identified estimating maximal aerobic power of rugby players: Multistage fitness, Yo-Yo intermittent recovery level 1 test, 30–15 intermittent fitness (30–15_IFT_) and the 1500 m run. The multistage fitness was commonly used in a number of studies [7, 8, 10, 16, 27, 30–37, 40, 49, 50, 61–64]. However, there is paucity of information on the measurement properties for maximal aerobic power in rugby or related sports. Only one study of “good” methodological quality assessed the reliability and the usefulness of the 30–15 intermittent fitness in rugby participants [68]. Best evidence synthesis showed moderate evidence to support the test-retest reliability of the 30–15 Intermittent Fitness test. There were no high-quality studies providing evidence on the measurement properties of tests identified for measuring anaerobic endurance such as the T120 s, Wingate 60 cycle, 300 m Shuttle Run and the 400 m Sprint tests. Holloway et al. [70] evaluated the validity of the T120 s test and compared the validity of the test to the Wingate 60 cycle test. According to the COSMIN guidelines, the study was rated as of “poor” methodological quality as the study had 12 participants.

There were number of studies that evaluated agility/change of direction speed of rugby players. There tests commonly used included: 505 test, Modified 505 test, Illinois Agility test, Change of Direction Speed test and Agility test [6, 16, 19, 32, 34, 35, 40, 53, 74, 77]. There were no high-quality studies evaluating the measurement properties of these tests in rugby. This is despite the importance of agility as a physiological skill in the sport of rugby. There was only one study of “fair” methodological quality according to the COSMIN guidelines that evaluated the measurement properties of the 505 test, modified 505 test, and the L run test. The study showed positive rating for the test-retest reliability of these three agility tests. However, there was negative rating for the known group validity for these tests. These findings support best evidence synthesis results indicating that there is limited evidence on the reliability and construct validity of these tests in assessing agility of rugby players. There is still need for further high-quality studies evaluating the measurement properties of these tests in rugby players.

Lower-body muscular power was the second most commonly studied physiological characteristic among rugby players in the studies included in this review. Although, there were three tests identified estimating the lower-body muscular power in the included studies. We found no studies evaluating the measurement properties of all three tests in rugby. Evidence on the measurement properties were found in one “fair” study evaluating the intra/inter-reliability and criterion validity of the Vertical Jump test among soccer players. A positive rating was found for the intra/inter-reliability of the test. Evidence on criterion validity was found to be questionable (Table 8) as there was no convincing argument that the gold standard test used was “gold”. Overall, best evidence synthesis indicates limited level of evidence for the inter/intra-rater reliability and criterion validity of the Sergeant (vertical) jump test.

There were also no clinimetric studies found testing the measurement properties of tests for lower-body muscular strength, upper-body muscular strength and power. However, one study of fair methodology provided the evidence on the known-group validity of two tests of upper-body muscular endurance (bench press-repetitions-to-fatigue test at 60 kg and 102.5 kg). Best evidence synthesis indicates that there is limited evidence to support the validity of these two tests in evaluating upper-body strength-endurance.

### Limitations

The results of this review paper should be interpreted with the understanding of a number of important limitations. Currently, there are no published reviews investigating measurement properties of performance-based tests measuring physiological characteristics in rugby. This renders comparisons with other review studies impossible. However, it suffices to suggest that these results expose a research gap on high-quality studies evaluating measurement properties for physiological tests commonly used in rugby. Although it could also be a major strength for this review, the inclusion criteria only considered full-text peer reviewed articles and completely excluded grey literature. This publication bias likely threatens internal validity of results obtained on measurement properties for this review as unpublished studies are more likely to report negative or unfavourable results. Although the COSMIN has been developed for the evaluation of measurement properties and has been generally used in the literature for that purpose, the guidelines appear well-suited and more applicable for appraising the quality of questionnaire-based studies. In the context of performance-based tests such as used in rugby, the applicability of the COSMIN as a quality rating tool for the studies on measurement properties still requires careful consideration.

## Conclusion

This review identified 15 physiological characteristics commonly evaluated among rugby players. These include speed, repeated sprint and effort ability, repeated high-intensity exercise performance, prolonged high-intensity intermittent running ability, endurance, anaerobic endurance, maximal aerobic power and speed, agility, lower-body muscular power and strength, upper-body muscular strength and power and upper-body muscular endurance. The majority of these physiological characteristics had multiple tests for measurement. Overall, there is paucity of high-quality clinimetric studies evaluating measurement properties of commonly-used physiological tests in rugby. For those tests that had evidence on measurement properties, there was no test which was evaluated with respect to all measurement properties. More studies are required evaluating the measurement properties of the physiological tests commonly used in the sport of rugby. The 30–15 intermittent fitness test (30–15_IFT_) test was the best rated test on maximal aerobic power with moderate evidence supporting its test-retest reliability. The 5 m, 10 m and 20 m speed test were the best tests assessing speed, however, with limited evidence supporting their test-retest reliability and the known-group validity. The 505 test, Modified 505 test and Lrun tests were the best tests for measuring agility but with limited evidence supporting their test-retest reliability. The Vertical jump test was the best test for assessing lower-body muscular power, however, with limited level of evidence for inter-rater, intra-rater reliability and criterion validity. Furthermore, there is limited evidence on the known group validity of the upper-body strength endurance tests of Bench-Press repetitions-to-fatigue at 60 and 102.5 kgs.

## Additional files

Additional file 1: Stage 1 search strategy designed for Medline via PubMed. (DOCX 14 kb)

Additional file 2: Stage 2 search strategy designed for Medline via PubMed. (DOCX 14 kb)

## Abbreviations

1RM: One repetition maximum
30–15_IFT_: 30–15 intermittent fitness test
AFL: Australian Rules Football
CI: Confidence interval
CINAHL: Cumulative Index of Nursing and Allied Health
CMJ: Countermovement jump
CODS: Change of direction speed
COSMIN: Consensus-based Standards for the Selection of health Measurement Instruments
CV: Coefficient of variation
ES: Effect size
GPS: Global positioning system
HIEP: High intensity exercise performance
HIRA: High intensity running ability
HREC: Human Research Ethics Committee
ICC: Intraclass correlation coefficient
JS: Jump squat
MSF: Multistage fitness test
PRISMA: Preferred reporting items for systematic reviews and meta-analyses
RHIE: Repeated high intensity exercise
RL: Rugby league
RS^2^: Rugby specific repeated sprint test
RU: Rugby union
SEM: Standard error of measurement
T120S: Triple 120 m shuttle run test
TE%: Percent typical error
TEM: Typical error of measurement
U: Under
VJ: Vertical jump test
W60: Wingate 60s cycle test
Yo-Yo IRT1: Yo-yo intermittent recovery test level 1
Yo-Yo IRT2: Yo-yo intermittent recovery test level 2
