# Supplementary material for: A systematic review investigating measurement properties of physiological tests in rugby
Source: BMC Sports Sci Med Rehabil. 2017 Dec 28;9:24. doi: 10.1186/s13102-017-0081-1 (PMC5745687; doi:10.1186/s13102-017-0081-1)
Supplement: Supplementary file 1 — Stage 1 search strategy designed for Medline via PubMed. (DOCX 14 kb) [file 13102_2017_81_MOESM1_ESM.docx]

**Additional file 1: Search strategy**

**Stage 1: Medline via PubMed**

*((((speed OR sprint* OR acceleration OR momentum OR linear speed OR velocity OR repeated sprinting OR sprinting force OR repeated sprinting abilit* OR speed endurance OR running OR running speed OR prolonged high intensity intermittent running ability OR high intensity running OR aerobic power OR aerobic capacity OR maximal aerobic power OR anaerobic endurance OR anaerobic endurance fitness OR change of direction OR change of direction speed OR ability OR power OR muscular power OR explosive power OR strength OR muscular strength OR lower body muscular strength OR upper body muscular strength OR lower body muscular power OR upper body muscular power OR muscular endurance OR upper body muscular endurance OR flexibility))*

*AND*

*(adult OR senior OR adolescent* OR youth OR teenager* OR elite OR sub-elite OR male* OR under 13 players OR U13 OR under 14 players OR U14 OR under 15 players OR U15 OR under 16 players OR U16 OR under 17 players OR U17 OR under 18 players OR U18 OR under 19 players OR U19 OR under 20 players OR U20 OR first grade OR second grade OR age category OR age group OR professional OR semi-professional OR amateur OR boy OR junior OR athletes OR forwards OR backs OR positional differences OR playing position))*

*AND*

*(rugby OR rugby union OR rugby union team OR rugby league OR rugby player* OR elite OR sub-elite OR rugby players OR collision sport* OR talented OR talent identification OR talent selection OR player assessment OR player development OR non-talented OR draft* OR non-draft* OR skilled players OR non-skilled players OR starters OR non-starters OR positional differences OR collision sport OR intermittent sport OR contact sport))*

*AND*

*(physical OR physical skill* OR physical characteristic* OR physical fitness OR physical qualities OR physical demands OR physical abilities OR motor skill* OR motor abilities OR motor component OR motor performance OR movement characteristic* OR performance analys* OR performance OR performance standards OR physiological OR physiological characteristic* OR physiological variable* OR physiological capacities OR physiological demands OR physiological testing OR fitness measures OR fitness profile OR fitness test)*
