# Supplementary material for: A systematic review investigating measurement properties of physiological tests in rugby
Source: BMC Sports Sci Med Rehabil. 2017 Dec 28;9:24. doi: 10.1186/s13102-017-0081-1 (PMC5745687; doi:10.1186/s13102-017-0081-1)
Supplement: Supplementary file 2 — Stage 2 search strategy designed for Medline via PubMed. (DOCX 14 kb) [file 13102_2017_81_MOESM2_ESM.docx]

**Additional file 2**

**Stage 2: Medline via PubMed**

*(((psychometrics OR psychometric property* OR clinimetr* OR clinometr* OR clinimetric property* OR measurement property OR measurement* OR measuring OR reproducib* OR reproducibility of results OR reliab* OR test-retest OR intra-rater OR inter-rater OR measurement error OR standard error of measurement OR technical error of measurement OR typical error of measurement OR sensitiv* OR responsive* OR interpretab* OR meaningful change OR minimal important change OR minimal important difference OR minimal detectable change OR minimal detectable difference OR ceiling effect OR floor effect OR valid* OR construct valid* OR face valid*or validation OR discriminative validity OR concurrent valid* OR convergent valid*))*

*AND*

*((vertical jump test* OR Countermovement jump test OR Jump squat test OR Plyometric power system OR speed test* OR sprint test OR Linear speed test OR 5m speed test OR 5m sprint test OR 10m sprint test OR 10m speed test OR 15m sprint test OR 20m sprint test OR 20m speed test OR 40m sprint test OR 40m speed test OR 50m speed test OR 50m sprint test OR 60m speed test OR 60m sprint test OR repeated 20m sprint test* OR rugby specific repeated speed test OR repeated effort ability test OR repeated high-intensity exercise performance test OR repeated 12s sprint shuttle speed test OR 5-m run test OR multistage fitness stage test* OR 20m multistage shuttle run test OR L-run test* OR 505 test OR Agility 505 test OR Illinois agility test OR modified 505 test OR Change of direction speed test OR Agility test OR yo-yo intermittent recovery (level 1) test* OR yo-yo intermittent recovery (level 2) test OR 30-15 Intermittent fitness test OR 1500m run OR 1500m run metabolic fitness index OR Triple 120m shuttle test OR Wingate 60 cycle test OR 300m shuttle run test OR 1 repetition maximum bench press test* 1 repetition maximum chin up test OR 3 repetition maximum bench press OR 1 minute push up test OR 1RM bench press OR 1RM prone row OR 60s push up test OR 60s chin up test OR 60s Sit Up OR pull up test OR 20s chin up test OR 2kg medicine ball chest throw test OR bench throw OR overhead medicine ball throw OR 1rm back squat OR 1 repetition maximum back squat OR one repetition bench press repetitions to fatigue at 60kg and 102.5kg test OR pull-up test OR body mass bench press with repetition)))*

*AND*

*((rugby OR rugby union OR rugby union team OR rugby league OR rugby player* OR elite OR sub-elite OR rugby players OR collision sport* OR talented OR talent identification OR talent selection OR player assessment OR player development OR non-talented OR draft* OR non-draft* OR skilled players OR non-skilled players OR starters OR non-starters OR positional differences))*

*AND*

*((Australian Rules football OR Australian football OR collision sport OR intermittent sport OR contact sport OR team sports OR soccer OR football OR American football OR Gaelic football OR collision sport))*.
